# Type‐I Heterostructure CdZnS/ZnS Core/Shell Quantum Dots Scintillators for Stable, High‐Resolution, and Real‐Time X‐Ray Imaging

**DOI:** 10.1002/advs.202515465

**Published:** 2025-10-06

**Authors:** Ouyang Wang, Fei Zhang, Rui Zhang, Meng Wang, Wenqing Liang, Zhifeng Shi, Xinjian Li

**Affiliations:** ^1^ Key Laboratory of Material Physics of Ministry of Education School of Physics Zhengzhou University Daxue Road 75 Zhengzhou 450052 China; ^2^ School of Flexible Electronics (SoFE) Henan Institute of Flexible Electronics (HIFE) Henan University 379 Mingli Road Zhengzhou 450046 China

**Keywords:** CdZnS/ZnS quantum dots scintillators, core/shell structures, real‐time X‐ray imaging, stability

## Abstract

Real‐time X‐ray imaging plays a critical role in medical diagnostics (e.g., cardiovascular and pulmonary monitoring), nondestructive evaluation, and in situ investigations of dynamic material processes. However, commonly used scintillators in medical imaging, CsI(Tl), suffer from an intrinsically long decay time (> 100 ms), which severely limits their suitability for high‐temporal‐resolution dynamic imaging. Herein, this study systematically employs surface defect passivation and carrier non‐radiative recombination suppression strategies to successfully construct Cd_0.27_Zn_0.73_S/7 ML‐ZnS core/shell quantum dots (QDs) with a type‐I band alignment. Such QDs exhibit ultrahigh photoluminescence quantum yield of over 96%, ultrafast carrier recombination dynamics with a decay time of 1.15 ns, and outstanding chemical stability. By innovatively applying anodic aluminum oxide templates to induce nano‐confinement effects, ordered assembly and directional emission control of the QDs are achieved within nanopore arrays, achieving a spatial resolution of up to 12.04 lp mm^−1^. Leveraging this engineered scintillation platform, a high‐performance real‐time X‐ray imaging system with a frame rate of 60 fps (2 K resolution) is further developed. Compared to traditional computed tomography and magnetic resonance imaging technologies, this system achieves significant improvement in temporal resolution, enabling effective capture of dynamic information from transient physiological processes.

## Graphical abstract

Ultra‐bright Cd_0.27_Zn_0.73_S/7 ML‐ZnS core/shell quantum dots exhibit a photoluminescence quantum yield exceeding 96% and a fourfold enhancement in radioluminescence intensity compared to CsPbBr_3_. Variable‐temperature photophysical analysis reveals that the narrowband emission originates from direct recombination of edge excitons. These quantum dots feature nanosecond‐scale exciton lifetimes and demonstrate exceptional thermal, radiative, and aqueous stability under X‐ray exposure. Furthermore, integration with anodic aluminum oxide templates enables high‐resolution imaging of dynamic targets.

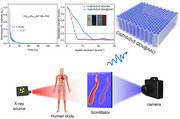

## 1 Introduction

Dynamic X‐ray imaging is crucial for capturing real‐time structural and functional changes, which find broad applications in medical diagnostics (e.g., cardiovascular and respiratory monitoring), nondestructive testing, and in situ studies of material dynamics.^[^ ^1^, ^2^, ^3^, ^4^, ^5^, ^6^ ^]^ Real‐time X‐ray imaging demands scintillators with the following critical attributes: 1) ultrafast radioluminescence (RL) decay dynamics to suppress motion‐induced image artifacts; 2) excellent structural integrity and radiation tolerance to maintain long‐term operational stability under continuous X‐ray exposure; 3) high intrinsic light yield and efficient photon output, which are essential for achieving sufficient image brightness and contrast at low X‐ray doses, particularly under high‐frame‐rate acquisition conditions. Although commercial scintillators like CsI(Tl) offer a high light yield (54 000 photons MeV^−1^) ideal for static imaging, their slow decay time (up to 100 ms) and hygroscopic nature limit their performance in dynamic X‐ray imaging and harsh environments.^[^ ^7^, ^8^, ^9^, ^10^, ^11^, ^12^ ^]^ While BGO scintillators exhibit a short decay time, their relatively low light yield makes them unsuitable for dynamic X‐ray imaging.^[^ ^7^, ^9^, ^13^ ^]^ These inherent limitations severely constrain their suitability for real‐time X‐ray imaging. Therefore, the identification of suitable scintillator materials is crucial for advancing dynamic X‐ray imaging.

Recently, metal halide perovskites CsPbBr_3_ quantum dots (QDs) have revived worldwide interest due to their efficient luminescence, short decay lifetime, high radiation hardness, and low‐temperature synthesis.^[^ ^14^, ^15^, ^16^ ^]^ Unfortunately, such QDs suffer from an inevitable photon self‐absorption problem owing to the free exciton recombination mechanism, resulting in a reduced light yield limited to 21 000 photons MeV^−1^.^[^ ^15^ ^]^ Furthermore, their insufficient stability poses a critical limitation to their further development and practical applications. The incorporation of CsPbBr_3_ as an emissive center within other organic luminescent matrices, combined with the construction of heterojunction architectures, can effectively enhance its photoluminescence quantum yield (PLQY).^[^ ^8^, ^15^, ^17^, ^18^ ^]^ Nevertheless, these strategies remain inadequate to fundamentally mitigate the intrinsic thermodynamic and environmental instability of the CsPbBr_3_ system.

In contrast, II‐VI semiconductors, CdZnS scintillators synthesized via solution process, exhibit nanosecond‐scale decay lifetimes and intrinsic exciton recombination comparable to CsPbBr_3_ QDs. However, the practical exploitation of these superior intrinsic properties in QD systems necessitates the efficient suppression of non‐radiative recombination pathways (such as Auger recombination), which are predominantly governed by surface‐related trap states; thus, meticulous surface and interface engineering is essential to preserve radiative efficiency.^[^ ^19^, ^20^, ^21^, ^22^ ^]^ By surface/interface engineering, the Cd_x_Zn_1−x_S/ZnS QDs show high stability and high PLQY in the blue spectral region. These properties enable efficient X‐ray‐to‐visible light conversion, making them promising candidates for high‐resolution X‐ray imaging. Based on this, by tuning their composition and integrating type I heterostructure engineering, their luminescent performance can be further enhanced for dynamic biomedical imaging applications.^[^ ^23^, ^24^, ^25^, ^26^ ^]^

In this work, for the first time, high‐performance Cd_x_Zn_1‐x_S/y ML‐ZnS core/shell QDs scintillators were developed via the hot‐injection method, achieving a PLQY exceeding 96% through effective surface defect passivation and suppression of non‐radiative recombination pathways. The materials exhibit exceptional radiation tolerance, maintaining structural and optoelectronic integrity under elevated temperatures and high‐dose irradiation conditions. To construct low‐scattering scintillator screens, the QDs were integrated into anodic aluminum oxide (AAO) templates, enabling high‐resolution dynamic imaging of simulated vascular architectures, owing to the short X‐ray afterglow decay time of 1.15 ns. The system supports dynamic imaging at 60 frames per second (fps) with high spatiotemporal resolution, as demonstrated by blur‐free tracking of a steel ball moving at 20 mm s^−1^. The capability to resolve metallic filaments and visualize simulated thrombus flow underscores its promise for clinical dynamic vascular imaging and real‐time micromotion tracking applications. This work establishes a robust platform of high‐performance QD scintillators, enabling ultrafast, high‐resolution dynamic imaging and paving the way for real‐time visualization and precise monitoring in advanced biomedical applications.

## 2 Results and Discussion

**Figure 1:**
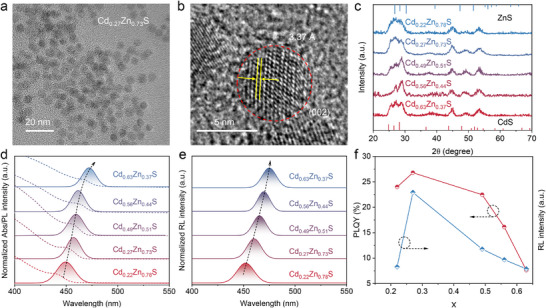
a) TEM image and b) the HRTEM image of Cd_0.27_Zn_0.73_S QDs. c) XRD patterns of Cd_x_Zn_1−x_ S QDs at different values of *x*. d) The PL spectra, e) RL spectra, and f) variation of PLQY and RL intensity of Cd_x_Zn_1‐x_S QDs at different values of x.

Cd_x_Zn_1−x_S and Cd_x_Zn_1−x_S/ZnS QDs were synthesized via a conventional hot‐injection method, as illustrated in Figure S1 (Supporting Information). Precise modulation of the feeding ratios of Cd(OA)_2_ and Zn(OA)_2_ enables fine control over the Cd^2+^ incorporation into the ZnS lattice, thereby facilitating the formation of Cd_x_Zn_1−x_S alloyed QDs with tunable stoichiometry. We used inductively coupled plasma‐optical emission spectroscopy (ICP‐OES) to analyze the compositional variations in the core QDs, which revealed Cd^2+^ contents of 22%, 27%, 49%, 56%, and 63% in five representative samples (Figure S2, Supporting Information). Transmission electron microscopy (TEM) images demonstrate that all QDs exhibit a uniform (**Figure** **1**a; Figure S3, Supporting Information) spherical morphology with a narrow size distribution of 5.4 ± 0.4 nm, irrespective of the Cd^2+^ doping concentrations. High‐resolution TEM further confirms a gradual increase in interplanar lattice spacing as Cd^2+^ concentration increases (Figure 1b; Figures S3 and S4, Supporting Information), which is attributed to the larger ionic radius of Cd^2+^ (0.95 Å) compared to Zn^2+^ (0.74 Å), which is consistent with the results of X‐ray diffraction (XRD) measurements shown in Figure 1c. Notably, a gradual shift of the (002) diffraction peaks toward lower angles is observed with increasing Cd^2+^ concentration, in accordance with Vegard's law.^[^ ^27^, ^28^, ^29^ ^]^ Moreover, no impurity phases were observed in the XRD patterns, indicating that Cd^2+^ ions are homogeneously incorporated into the ZnS lattice, forming a solid solution without phase segregation. These findings collectively confirm the successful formation of alloyed Cd_x_Zn_1−x_S QDs with tunable composition and well‐defined crystal structure.

To comprehensively investigate the influence of Cd^2+^ content on the luminescent behavior of Cd_x_Zn_1−x_S QDs under different energy excitations, we systematically analyzed their photoluminescence (PL) and radioluminescence (RL) spectra irradiated by UV and X‐ray. As shown in Figure 1d,e, the PL and RL spectra of the QDs exhibit highly consistent spectral profiles across varying Cd^2+^ doping levels, including similar peak positions and emission line shapes. This strong spectral overlap suggests that both UV and X‐ray excitation invoke the same fundamental photophysical mechanisms. Specifically, the emission spectra under such excitation sources feature narrow full width at half maximum (FWHM < 30 nm) and symmetric Gaussian‐like peak shapes—hallmarks of radiative recombination dominated by single exciton states. These observations align well with theoretical predictions for band‐edge exciton recombination,^[^ ^30^ ^]^ confirming that excitonic emission from the band edge is the primary luminescence pathway for Cd_x_Zn_1‐x_S QDs, regardless of excitation modality. Notably, a systematic redshift in the emission peak is observed with increasing Cd content (x), which can be attributed to the relatively weaker coordination ability of oleic acid (OA) toward Cd^2+^ compared to Zn^2+^. This weaker binding enhances the reactivity of Cd(OA)_2_ with sulfur precursors during synthesis, resulting in QDs with a radial compositional gradient—characterized by a CdS‐rich core even at high Zn/Cd feed ratios.^[^ ^31^ ^]^

Figure 1f presents the variation in PLQY and RL intensity of Cd_x_Zn_1−x_S QDs as a function of Cd^2+^ doping concentration (x). The highest PLQY of 26.8% and the maximum RL intensity are both achieved at *x* = 0.27, indicating an optimal compositional regime. This trend suggests that moderate Cd^2+^ incorporation enhances the radiative recombination efficiency by passivating surface or lattice defects and promoting band‐edge exciton emission. However, excessive Cd^2+^ doping likely introduces structural disorder or defect states that facilitate non‐radiative pathways, thereby quenching luminescence.^[^ ^32^ ^]^ The peak performance observed at *x* = 0.27 thus represents a critical balance between radiative and non‐radiative processes, where compositional tuning most effectively optimizes the photophysical properties. Based on this optimization, the Cd_0.27_Zn_0.73_S was selected for subsequent core/shell structure fabrication to further improve the optical performance and stability of the QDs.

**Figure 2:**
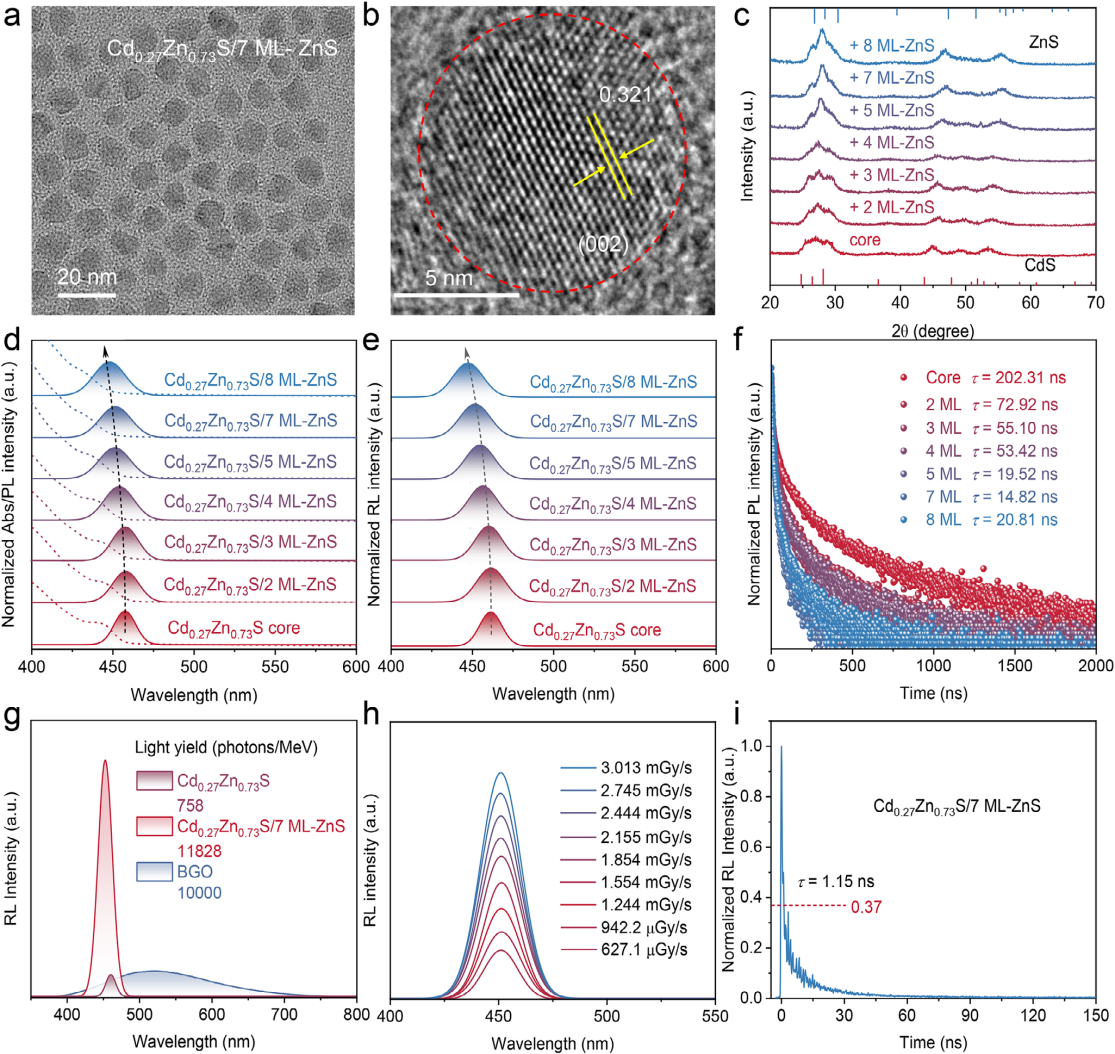
a) TEM image and b) the HRTEM image of Cd_0.27_Zn_0.73_S/7 ML‐ZnS QDs. c) XRD patterns, d) UV–vis and PL spectra, e) RL spectra, and f) PL decay dynamics of Cd_0.27_Zn_0.73_S/y ML‐ZnS QDs with different shell thicknesses. g) RL spectra of Cd_0.27_Zn_0.73_S films, Cd_0.27_Zn_0.73_S/7 ML‐ZnS, and BGO (dose rate: 50.87 µGy s^−1^; voltage: 50 kV). h) RL spectra of Cd_0.27_Zn_0.73_S/7 ML‐ZnS film under X‐ray radiation at different dose rates. i) RL decay dynamics of Cd_0.27_Zn_0.73_S/7 ML‐ZnS QDs.

To improve the luminescent properties, a type‐I heterostructure design was proposed to facilitate carrier confinement and boost radiative recombination efficiency. In this context, Cd_0.27_Zn_0.73_S QDs were employed as the core for ZnS shell epitaxial growth to form a core‐shell type‐I heterostructure. As illustrated in Figure S1 (Supporting Information), ZnS shell layers were epitaxially deposited by hot‐injecting Zn(OA)_2_ precursors at 310 °C after the formation of Cd_0.27_Zn_0.73_S cores. The shell thickness was precisely controlled by adjusting the reaction time to achieve tailored core/shell architectures. Structural characterizations confirm the successful formation of the ZnS shell. TEM analysis reveals a systematic increase in QD diameter from 5.47 to 10.34 nm with increasing shell thickness (**Figure** **2**a; Figures S5 and S6, Supporting Information), indicating effective shell growth. HRTEM images show clear lattice fringes extending across the entire nanocrystal (Figure 2b; Figure S5f, Supporting Information), verifying the high crystallinity of the core/shell QDs. Notably, the interplanar spacing progressively decreases with increasing shell thickness, in line with the smaller lattice constant of ZnS (*a* = 3.82 Å) relative to CdS (*a* = 4.14 Å), confirming the coherent epitaxial growth of ZnS on the CdZnS core.^[^ ^33^ ^]^ Elemental mapping via TEM‐EDS further corroborates the core/shell structure (Figure S7, Supporting Information). Cd signals are predominantly localized within the QD core region, while Zn and S are uniformly distributed across the entire QD, clearly distinguishing the shell domain.

XRD analysis reveals characteristic peaks of the wurtzite structure for all samples (Figure 2c). With increasing ZnS shell thickness, the diffraction peaks of the Cd_0.27_Zn_0.73_S/y monolayer‐ZnS (Cd_0.27_Zn_0.73_S/y ML‐ZnS, y is the number of monolayers of the ZnS shell, *y* = 0, 2, 3, 4, 5, 7, 8) QDs gradually shift toward higher 2θ values, located between those of wurtzite CdS and ZnS references. In particular, the (002) diffraction peak shows a continuous shift toward higher angles as the shell grows, consistent with the trend observed in HRTEM where the interplanar spacing decreases—further supporting epitaxial ZnS shell formation. X‐ray photoelectron spectroscopy (XPS) measurements show a significant attenuation of Cd 3d signal intensity upon shell growth (Figure S8, Supporting Information), indicating that Cd is largely confined to the core region, while the ZnS shell effectively suppresses surface Cd signal detection. The influence of ZnS shell thickness on the optical properties of Cd_0.27_Zn_0.73_S core QDs was systematically investigated. UV–vis absorption spectra reveal a gradual blue shift in the absorption edge with increasing shell thickness (Figure 2d). Tauc plot analysis shows that the optical bandgap increases from 2.734 to 2.805 eV with 8 ML of ZnS shell growth (Figure S9, Supporting Information), indicating enhanced quantum confinement effects. Interestingly, both PL and RL spectra (Figure 2d,e) exhibit the same spectral blue shift, which is ascribed to thermally induced diffusion of Zn^2+^ into the core during high‐temperature shell growth,^[^ ^34^ ^]^ leading to non‐uniform alloying—a phenomenon consistent with the observed bandgap widening.

Optical performance analysis reveals a non‐monotonic dependence of PLQYs and RL intensity on shell thickness (Figure S10, Supporting Information). The QDs exhibit optimal luminescent performance at a shell thickness of 7 ML, with maximum RL intensity and a peak PLQY of 96.75%, which indicates that the surface defects of quantum dots have been effectively passivated, suppressing defect‐related radiative or non‐radiative recombination. Time‐resolved PL spectra (Figure 2f) further show that the average PL lifetime gradually decreases to 14.82 ns at 7 ML. Notably, the proportion of the fast radiative decay component (*τ*_1_) increases and stabilizes with thicker shells, indicating effective exciton confinement. Meanwhile, the decay time of the slower component (*τ*_2_) shortens, confirming the passivation of surface trap states by the ZnS shell. However, when the shell thickness exceeds 7 ML, the average PL lifetime anomalously increases to 19.52 ns, accompanied by a significant extension of the *τ*_2_ component (Figure S11, Table S1, Supporting Information). This is attributed to the formation of new non‐radiative recombination centers caused by lattice mismatch‐induced strain in overly thick shells.^[^ ^35^ ^]^ This mechanism provides a rational explanation for the bell‐shaped dependence of PLQY and RL intensity on shell thickness.

Based on the above optimization, the Cd_0.27_Zn_0.73_S/7 ML‐ZnS core/shell QDs exhibit optimal RL intensity and radiative recombination efficiency. To systematically evaluate their scintillation performance, we first analyzed their intrinsic X‐ray absorption characteristics. As shown in Figure S12, the X‐ray absorption coefficient of the Cd_0.27_Zn_0.73_S/7 ML‐ZnS core/shell material is comparable to that of binary ZnS and CdS. However, due to its lower average atomic number, the overall absorption is correspondingly weaker, in agreement with theoretical expectations. To enable an objective comparison of scintillation performance, we prepared standard samples of Cd_0.27_Zn_0.73_S core QDs, Cd_0.27_Zn_0.73_S/7 ML‐ZnS core‐shell QDs, and CsPbBr_3_ QDs, each with identical dimensions (2 mm thickness, 10 mm diameter).

As depicted in Figure 2g, by comparing with BGO (10 000 photons MeV^−1^), the core‐shell QDs reach a light yield of 11 828 photons MeV^−1^, which is 15.6‐fold higher than that of 758 photons MeV^−1^ of the core QDs. This significant improvement arises from two synergistic effects: 1) effective surface defect passivation by the ZnS shell, which elevates the PLQY to 96.75% and substantially suppresses nonradiative recombination channels; and 2) the formation of a type‐I band alignment at the core‐shell interface, which enhances carrier confinement.^[^ ^36^, ^37^ ^]^ Furthermore, dose‐dependent RL measurements (Figure 2h; Figure S13, Supporting Information) show a highly linear response of the RL intensity to X‐ray dose (*R* ^2^ = 0.997), with no observable saturation or quenching effects. This behavior rules out defect‐assisted emission pathways and confirms that the scintillation originates from direct band‐edge exciton recombination. Besides, as shown in Figure S14 (Supporting Information), the detection limit (DL) of Cd_0.27_Zn_0.73_S core QDs films is 793.2 nGy s^−1^, which is much lower than that required for conventional Computed Tomography (5.5 µGy·s^−1^).^[^ ^38^ ^]^ Rapid scintillation decay is essential for capturing transient events in X‐ray biomedical imaging, where maintaining high temporal fidelity is critical for accurate dynamic visualization. Notably, such QD scintillators exhibit an ultrafast decay time of 1.15 ns (Figure 2i)—orders of magnitude shorter than under optical excitation—due to X‐ray‐induced ultra‐high carrier densities activating dominant non‐radiative processes like cross‐relaxation and energy transfer,^[^ ^39^ ^]^ which highlights their strong potential for high‐resolution and high‐speed X‐ray imaging applications.

**Figure 3:**
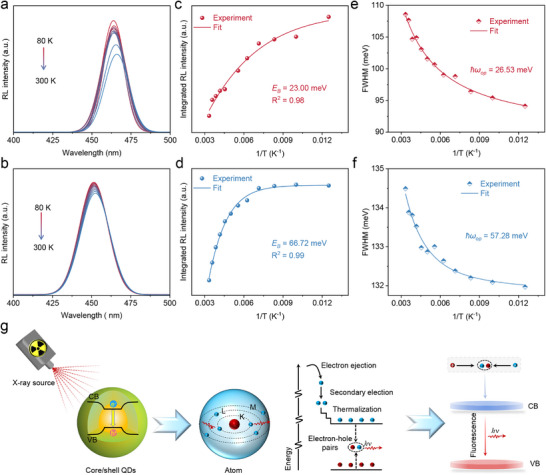
Temperature‐dependent RL spectra of a) the Cd_0.27_Zn_0.73_S core QDs and b) Cd_0.27_Zn_0.73_S/7 ML‐ZnS core/shell QDs composites taken from 80 to 300 K. The relationship between the integrated RL intensity and the reciprocal temperature from 80 to 300 K for c) Cd_0.27_Zn_0.73_S core QDs and d) Cd_0.27_Zn_0.73_S/7 ML‐ZnS core/shell QDs. FWHM of e) Cd_0.27_Zn_0.73_S core QDs and f) Cd_0.27_Zn_0.73_S/7 ML‐ZnS core/shell QDs as a function of reciprocal temperature. g) Schematic representation of the X‐ray scintillation process for Cd_0.27_Zn_0.73_S/7 ML‐ZnS QDs emitters.

To gain a deeper understanding of the role of the ZnS shell in modulating the optoelectronic properties of Cd_0.27_Zn_0.73_S QDs, we systematically investigated the temperature‐dependent luminescence behavior of both the core (Cd_0.27_Zn_0.73_S) and core/shell (Cd_0.27_Zn_0.73_S/7 ML‐ZnS) QDs. Experimental results show that within the temperature range of 80–300 K, the core‐only QDs exhibit significant thermal quenching, with their RL intensity dropping to only 70% of the initial value at 300 K (**Figure** **3**a). This is primarily attributed to nonradiative recombination processes mediated by surface defect states. In contrast, the core/shell QDs demonstrate excellent temperature stability, maintaining 91% of their initial RL intensity even at 300 K (Figure 3b). This result directly confirms that the ZnS shell effectively passivates surface defects, significantly reducing the probability of nonradiative recombination. To quantitatively analyze the thermal quenching behavior, we calculated the exciton binding energy (*E*_B_) of the two materials using the Arrhenius model^[^ ^40^ ^]^:

(1) $$I\left(T\right)=\frac{I_{0}}{1+\mathrm{Aexp}\left(-\frac{E_{B}}{k_{B}T}\right)}$$

where, *I*_0_ is the emission intensity at 80 K, *A* is the fitted constant, and *k_B_* is the Boltzmann constant. As shown in Figure 3c,d, the *E*_B_ of Cd_0.27_Zn_0.73_S core QDs and Cd_0.27_Zn_0.73_S/7 ML‐ZnS core/shell QDs were determined to be 23.0 and 66.72 meV, respectively, through fitting analysis. This result is in excellent agreement with the temperature‐dependent PL spectra under UV excitation (Figure S15c,d, Supporting Information), further validating the significant role of the ZnS shell in modulating the excitonic properties of the QDs.

The enhanced excitonic behavior can be attributed to a combination of synergistic mechanisms: 1) The type‐I band structure formed by the ZnS shell significantly amplifies the quantum confinement effect of charge carriers; 2) The gradient composition of the CdZnS/ZnS interface effectively eliminates surface defects such as dangling bonds; 3) Lattice strain at the interface induces band renormalization, which further optimizes the exciton characteristics.^[^ ^37^, ^41^ ^]^ Notably, the exciton binding energy of 62.99 meV is considerably higher than the thermal energy at room temperature (*k*_B_T ≈ 26 meV), ensuring that the core/shell QDs maintain excellent exciton stability even in high‐temperature environments, thereby laying a solid foundation for their application in high‐temperature optoelectronic devices.

Both Cd_0.27_Zn_0.73_S core QDs and their core/shell structures exhibit a temperature‐dependent bandgap redshift of ≈70 meV within the 80–300 K temperature range (Figure S16, Supporting Information). This temperature‐dependent behavior can be accurately described by the Varshni empirical equation^[^ ^42^ ^]^:

(2) $$E_{g}\left(T\right)=E_{0}-\alpha \frac{T^{2}}{T+\beta }$$

where *E*_0_ is the energy gap at 0 K. α is the temperature coefficient, and the value of β is close to the Debye temperature of the material. The fitting results show that the Varshni parameters for both types of QDs are in excellent agreement with those reported for bulk materials in the literature (Table S2, Supporting Information),^[^ ^43^ ^]^ confirming that the luminescence is primarily derived from electron‐hole recombination at the Cd_0.27_Zn_0.73_S core. This finding not only validates that the temperature‐dependent bandgap shift is a consequence of lattice thermal expansion, which causes band contraction, but also provides insights from a band engineering perspective on how the core/shell structure modulates exciton properties.

In addition, the FWHM of the RL spectra for both types of QDs consistently broadens with increasing temperature (Figure 3e,f). To quantitatively analyze this temperature‐dependent broadening, we applied the independent Boson model^[^ ^44^ ^]^:

(3) $$\Gamma \left(T\right)=\Gamma_{0}+\sigma T+\Gamma_{\mathrm{op}}{\left(e^{\hbar \omega_{\mathrm{op}}/k_{B}T}-1\right)}^{-1}$$

where Γ_0_ represents the inhomogeneous broadening resulting from the size/compositional distribution of the QDs. Notably, the fitting results reveal that the optical phonon energy (*ħω_op_*) for Cd_0.27_Zn_0.73_S core QDs and core/shell structures are 26.53 and 57.28 meV, respectively, which are in good agreement with the PL spectral results (Figure S15e,f, Supporting Information). This suggests that the phonon excitation energy in the core/shell structure is enhanced by 116%. This significant enhancement is primarily attributed to the strong phonon confinement effect introduced by the ZnS shell and the modulation of interfacial stress, which collectively improve the optical stability of the core/shell QDs at elevated temperatures, thus providing critical support for their practical applications.

Figure 3g illustrates the RL mechanism of the type‐I band‐aligned Cd_0.27_Zn_0.73_S/7 ML‐ZnS core/shell QDs under X‐ray irradiation. Due to their type‐I heterostructure, where the ZnS shell effectively confines charge carriers within the Cd_0.27_Zn_0.73_ core, in the photoelectric effect and Compton scattering, the primary photon interacts with QD atoms and generates highly energetic secondary electrons. The ejected electrons go through the QDs and release many low‐energy free electrons by ionization. The free electron moves to the conduction band, leaving a hole in the valence band. The hole migrates to the top of the valence band, causing electrons to transfer to lower energy levels within the band. The electron that was elevated to the conduction band relaxes to the bottom by individual or collective actions. The resulting carrier recombination within the spatially confined type‐I structure leads to efficient RL emission centered at 460 nm, characteristic of the Cd_0.27_Zn_0.73_S/7 ML‐ZnS core/shell QDs.

**Figure 4:**
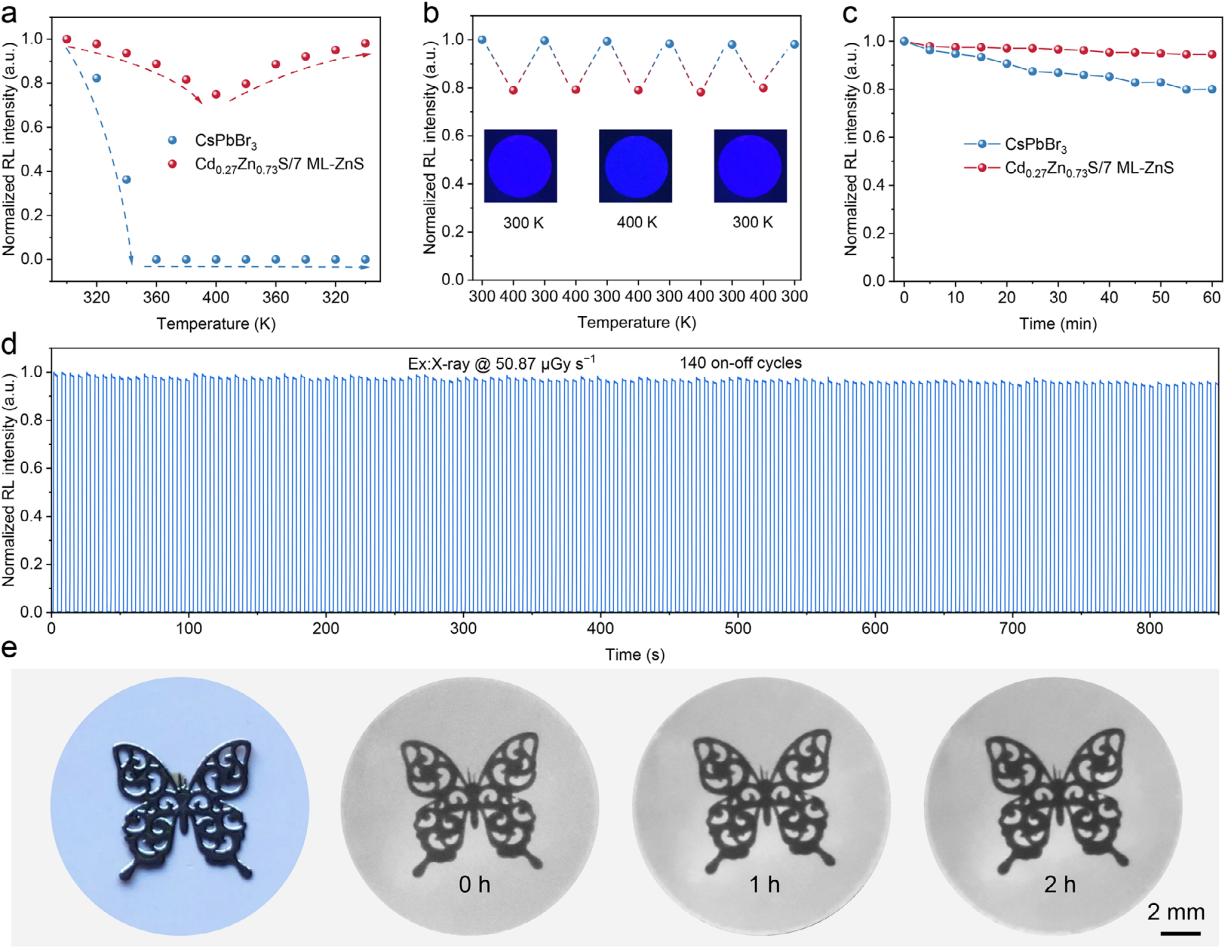
a) Evolution of integral RL intensities of CsPbBr_3_ core and Cd_0.27_Zn_0.73_S/7 ML‐ZnS core/shell QDs at different temperatures. b) Five consecutive thermal cycling tests of Cd_0.27_Zn_0.73_S/7 ML‐ZnS QDs (300–400 K). c) The RL stability of Cd_0.27_Zn_0.73_S/7 ML‐ZnS core/shell QDs and CsPbBr_3_ under continuous X‐ray irradiation. d) RL intensity of Cd_0.27_Zn_0.73_S/7 ML‐ZnS core/shell QDs under 140 on/off cycles of X‐ray irradiation (dose rate: 50.87 µGy_air_ s^−1^). e) Bright‐field (left) and X‐ray (right) images of the butterfly based on the Cd_0.27_Zn_0.73_S/7 ML‐ZnS film for different times under X‐ray irradiation, respectively.

Stability is a critical parameter for the commercialization of scintillator materials. Thus, we systematically evaluated the stability of Cd_0.27_Zn_0.73_S/7 ML‐ZnS core/shell QDs. Thermal stability tests demonstrated that the core/shell QD films exhibit excellent reversible thermal response characteristics in the 300–400 K temperature range (**Figure** **4**a). Even at 400 K, the RL intensity retained 75% of its initial value and could be fully restored to its original level upon cooling. In contrast, CsPbBr_3_ QDs suffered irreversible complete quenching at 360 K. CsPbBr_3_ QDs suffer from instability due to crystal defects, ion migration, environmental sensitivity, and phase transitions, which promote non‐radiative recombination and structural degradation.^[^ ^45^, ^46^, ^47^ ^]^ In contrast, Cd_0.27_Zn_0.73_S/7 ML‐ZnS core/shell QDs achieve high stability through effective surface passivation, a robust physical barrier, and stress regulation provided by the lattice‐matched ZnS shell.^[^ ^19^, ^37^, ^41^, ^48^, ^49^ ^]^ Notably, after five consecutive thermal cycles (300–400 K), the RL intensity of the core/shell QDs maintained 100% of its initial value (Figure 4b). And the X‐ray imaging resolution of the film showed minimal change during the thermal cycles (Figure S17, Supporting Information), demonstrating stable high‐resolution imaging performance even at 400 K.

Furthermore, radiation stability tests confirmed the superior performance of the core‐shell QDs. After 60 min of continuous X‐ray exposure at a dose rate of 50.87 µGy_air_·s^−1^, the RL intensity of the core/shell QDs decreased by less than 3%, significantly outperforming CsPbBr_3_ (which experienced a 20% decrease) (Figure 4c), and the spectral shape does not change significantly (Figure S18, Supporting Information). Even after 140 X‐ray switch cycles, the luminous intensity of the core/shell QDs remained stable (Figure 4d), and no significant change in imaging resolution was observed after 2 h of continuous irradiation (Figure 4e). Additionally, the material exhibited excellent environmental water stability. After soaking the QDs core/shell films in water for 4 h, their luminescent intensity remained unchanged (Figure S19, Supporting Information), and the water contact angle of 115.6° confirmed the outstanding hydrophobic properties of the material (Figure S20, Supporting Information). Furthermore, after one year of storage under ambient conditions (humidity: 30–60%, temperature: 20–35 °C), the structural integrity was maintained without the emergence of additional diffraction peaks (Figure S21, Supporting Information). These results demonstrate that Cd_0.27_Zn_0.73_S/7 ML‐ZnS core/shell QDs possess exceptional thermal stability, radiation stability, and environmental water stability. Their comprehensive performance significantly surpasses that of conventional CsPbBr_3_ QDs, showing great potential for applications in X‐ray imaging, underwater detection, and other fields.

**Figure 5:**
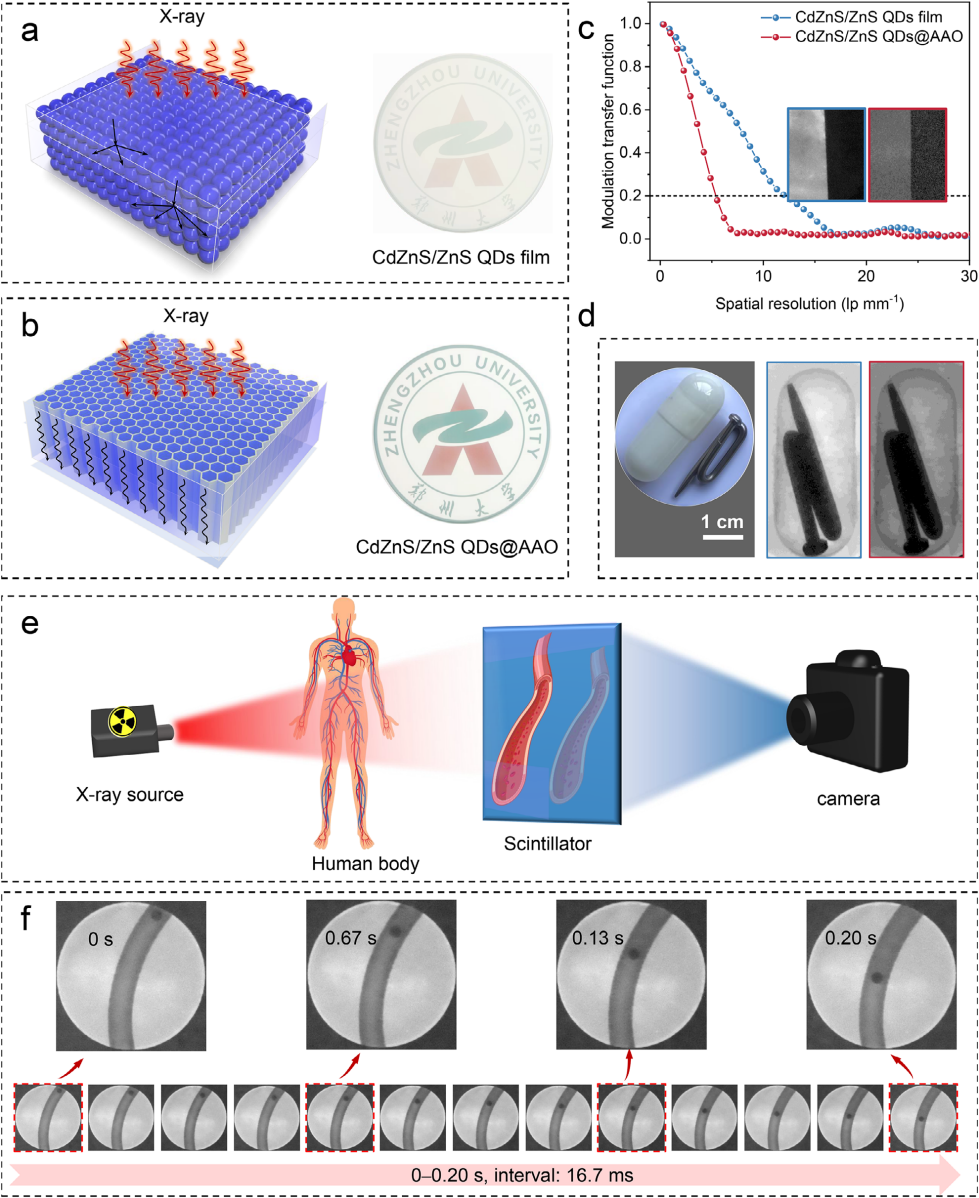
Scintillator screen schematic (left) and photograph (right) of a) Cd_0.27_Zn_0.73_S/7 ML‐ZnS QDs films and b) Cd_0.27_Zn_0.73_S/7 ML‐ZnS QDs@AAO films, which reduces light scattering and improves the spatial resolution. c) MTF curves with insets showing the corresponding X‐ray edge images of the QDs@AAO film (blue box) and QDs film (red box). d) X‐ray images of a metal screw embedded within an optically opaque capsule of the QDs@AAO film (blue box) and QDs film (red box) (dose rate: 50.87 µGy s^−1^; voltage: 50 kV; beam current: 20 µA; exposure time: 3 s). e) Schematic of the X‐ray imaging diagnosis for blood flow at the inferior vena cava. f) Dynamic X‐ray imaging at 60 frames per second under continuous X‐ray radiation (dose rate: 50.87 µGy s^−1^; beam current: 20 µA; exposure time, 16.7 ms).

To systematically evaluate the X‐ray scintillation performance of Cd_0.27_Zn_0.73_S/7 ML‐ZnS QDs, a comparative experimental design was employed in which QDs thin films were fabricated on both optical‐grade glass substrates and ordered porous AAO templates. Characterization was conducted using a custom‐built high‐resolution X‐ray imaging system comprising a microfocus X‐ray source, a precision sample stage, optical mirrors, and a high‐sensitivity CCD camera. The results reveal that QD films deposited on glass substrates exhibit significantly reduced spatial resolution due to pronounced multiple light‐scattering effects at grain boundaries and surface inhomogeneities (**Figure** **5**a). Quantitative analysis via the modulation transfer function (MTF) indicates that a 50 µm‐thick film achieves a spatial resolution of only 5.42 lp mm^−1^ at MTF = 20% (Figure 5c), which is inadequate for high‐definition imaging applications.

To overcome these limitations, we developed a vacuum‐assisted infiltration technique to confine the QDs within the vertically aligned nanopores of a 50 µm‐thick AAO template. SEM images clearly demonstrate the complete and uniform filling of the nanopores by QDs (Figures S22 and S23, Supporting Information), while EDS elemental mapping confirms a homogeneous distribution of Cd, Zn, and S within the AAO matrix—indicative of well‐organized QDs assembly within the confined environment (Figures S22c and S23c, Supporting Information). Meanwhile, we prepared large‐area scintillator films. Through planar and cross‐sectional analysis of multiple regions, including the sample center and edges, we confirmed that the quantum dot fillers exhibit high uniformity and integrity throughout the entire 50 µm pore depth and across the entire 25 mm diameter range (Figures S24–S27, Supporting Information). As shown in Figure S28 (Supporting Information), the AAO template can further enhance the thermal stability of the material under high‐temperature conditions. Moreover, the AAO‐based composite films exhibit excellent optical transparency in the visible spectrum. Optical photographs taken under ambient light further reveal high spatial uniformity and minimal scattering (Figure 5b). This unique confinement architecture significantly enhances imaging performance via two mechanisms: 1) the waveguiding effect of AAO nanopores suppresses lateral photon scattering, and 2) the ordered alignment of QDs improves emission homogeneity. As a result, the spatial resolution of the AAO‐based composite film is markedly improved, reaching 12.04 lp mm^−1^ at MTF = 20% (Figure 5c), thereby demonstrating the efficacy of nanoscale confinement in optimizing QD‐based scintillators for high‐resolution X‐ray imaging. To evaluate the imaging capability of the QDs@AAO system, a series of test objects was positioned between the scintillation screen and the X‐ray source. Notably, a metal screw embedded within an optically opaque capsule was distinctly resolved, highlighting the system's ability to capture fine structural details under challenging imaging conditions (Figure 5d).

Critically, dynamic X‐ray imaging is indispensable for capturing transient physiological processes, particularly in medical applications such as thrombus monitoring, where real‐time visualization of fluid dynamics and moving structures is essential.^[^ ^50^, ^51^ ^]^ Traditional dynamic imaging (e.g., conventional computed tomography (CT) and magnetic resonance imaging (MRI)) often fails to resolve rapid temporal changes and is prone to motion artifacts, potentially leading to inaccurate diagnoses of dynamic biological events.^[^ ^51^ ^]^ We thus developed a dynamic imaging system designed to simulate thrombus observation in a clinical context owing to the ultrafast decay time while maintaining high spatial resolution (Figure 5e; Figure S29, Supporting Information). Under continuous X‐ray irradiation at a dose rate of 50.87 µGy s^−1^, it successfully captures the motion of a steel ball at 60 fps (corresponding to a flow velocity of ≈20 mm s^−1^), significantly reducing motion blur and eliminating ghosting artifacts (Figure 5f; Movie S1, Supporting Information). As the dose rate was further reduced, the image became blurred (Figure S30, Supporting Information). A dose rate of 50.87 µGy s^−1^ was the lowest explored that maintained high resolution while complying with clinical safety standards. The system captured the movement of a steel ball (≈20 mm s^−1^ flow velocity). Quantitative metrics confirm that the system achieves a temporal resolution of 60 fps, effectively minimizes motion‐induced artifacts, and preserves consistent image contrast throughout rapid imaging sequences—performance parameters that meet or exceed clinical standards for dynamic vascular and hemodynamic imaging.^[^ ^52^, ^53^ ^]^ Such capabilities enable precise tracking of micro‐scale movements, offering transformative potential for in vivo diagnostics and mechanistic studies of dynamic processes.

## 3 Conclusion

We have demonstrated a type‐I heterostructure Cd_0.27_Zn_0.73_S/7 ML‐ZnS core/shell QDs with PLQY exceeding 96%, attributed to effective surface defect passivation and suppression of carrier non‐radiative recombination. Moreover, the combination of strong X‐ray emission, ultrafast decay time of 1.15 ns, and robust environmental stability under thermal stress, moisture exposure, and prolonged X‐ray irradiation renders these QDs highly promising for high‐temporal‐resolution real‐time X‐ray imaging. By employing an AAO template that guides light emission through core/shell Cd_0.27_Zn_0.73_S/7 ML‐ZnS‐filled nanopores, high‐resolution and high‐speed real‐time X‐ray imaging at 60 fps (2K resolution) is achieved, demonstrated by blur‐free tracking of a steel ball moving at 20 mm s^−1^. The capability to resolve fine metallic structures and dynamically visualize simulated thrombus flow demonstrates the system's strong potential for clinical real‐time vascular imaging and microscale motion tracking. This work establishes a highly stable and scalable QDs scintillator platform that enables ultrafast, high‐resolution dynamic X‐ray imaging, paving the way for advanced biomedical applications requiring real‐time visualization and quantitative monitoring of transient physiological processes.

## 4 Experimental Section

### Chemicals

Chemicals were used directly without any purification: sulfur powder (S, Sigma–Aldrich 99.998%), 1‐octadecene (ODE, Sigma–Aldrich 90%), oleic acid (OA, Sigma–Aldrich 90%), Cadmium oxide (CdO, Sigma–Aldrich 99.99%), zinc oxide (ZnO, Sigma–Aldrich 99.9%), Paraffin (analytical grade), acetone (analytical grade), hexanes (analytical grade) and methanol (analytical grade) were obtained from Tianjin Yongda Chemical Reagent Co., Ltd, China.

### Precursor Preparation

Sulfur stock solution was synthesized as follows: sulfur (12 mmol) and 1‐octadecene (24 mL) were mixed in a 100 mL three‐neck flask. The mixture was then degassed at 100 °C for 5–10 min. After this, the solution was heated to 150 °C under a N_2_ flow with vigorous stirring to get a clear solution.

### Cadmium oleate (Cd(OA)_2_) Solution Synthesis

Cadmium oxide (10 mmol), oleic acid (15 mL), and 1‐octadecene (35 mL) were placed in a 100 mL three‐neck flask. The mixture was then degassed at 150 °C for 5–10 min. After this, the solution was heated to 260 °C under a N_2_ flow with vigorous stirring to get a clear solution.

### Zinc oleate (Zn(OA)_2_) Solution Synthesis

Zinc oleate was prepared by mixing zinc oxide (20 mmol), oleic acid (30 mL), and 1‐octadecene (20 mL) in a 100 mL three‐neck flask. The mixture was then degassed at 150 °C for 5–10 min. After this, the solution was heated to 310 °C under a N_2_ flow with vigorous stirring until it became clear. Then the solution was cooled to room temperature and stored until further use.

### Cd_x_Zn_1‐x_S Core QDs Synthesis

0.3 mmol Cadmium oxide, 0.1 mmol Zinc oleate, 15 mL of paraffin oil, and 1 mL of oleic acid were placed in a 100 mL round flask. The mixture was heated to 150 °C, degassed under 0.1 Torr pressure for 20 min, filled with N_2_ gas, and further heated to 300 °C to form a clear mixture solution of Cd(OA)_2_ and Zn(OA)_2_. 0.4 mL of sulfur stock solution was rapidly added to the reaction flask at this temperature. To track the samples' PL spectra, they were extracted. After 10 min, the reaction was finished, the temperature was lowered to room temperature, and either methanol or acetone was used to purify the QDs. After being diluted with an excess of hexane, the resultant QDs were centrifuged four times using the precipitation/dispersion method with a solvent mixture of hexane/methyl alcohol (1/2 in volume ratio) under the same centrifugation conditions. The purified QDs were then re‐dispersed into hexane or chloroform for optical characterization, post‐treatments, and solid QD films.

### Cd_x_Zn_1‐x_S/ZnS Core/Shell QDs Synthesis

The ZnS shell was successively overcoated by adding a required amount of Zn(OA)_2_ and octanethiol dropwise into the reaction solution at a rate of 6 mL h^−1^ using a syringe pump (1.2 equivalent amounts refer to Zn(OA)_2_ diluted in 5 mL ODE). The solution underwent additional annealing at 310 °C for 3 h following the completion of the precursor infusion. Acetone or methanol was used to purify the QDs when the process was finished and the temperature was lowered to room temperature. Cd_x_Zn_1‐x_S/ZnS was performed like that of CdZnS.

### The Fabrication Process of Scintillator Screen

First, 50 µL of the abovementioned precursors were uniformly dropped on the 30 mm diameter round quartz vessel. Then, the empty AAO membranes were immersed in the precursors. Finally, transfer the AAO membranes immersed in the precursor solution to a vacuum drying oven for negative pressure filling at −70 kPa for 2 h. Subsequently, the CdZnS/ZnS Core/Shell QDs @AAO nano arrays were obtained.

### Characterization Section

The ICP‐OES data were obtained by the Agilent 5110 ICP‐OES. Room temperature UV–vis absorption was recorded with the utilization of the UV–vis absorption spectrometer SHIMADZU UV‐2600. The PLE and PL spectra, time‐resolved decay kinetics, excitation‐dependent PL responses, and temperature‐dependent emission profiles of PL spectra were comprehensively performed on the spectrometer HORIBA FluoroLog‐3. The system configuration incorporated an integrating sphere attachment for absolute quantum yield measurements and a computer‐controlled temperature stage for thermodynamic studies. The RL spectra and temperature‐dependent emission profiles of RL spectra were recorded with an Ocean Optics QE Pro spectrophotometer. The RL decay was collected by a photomultiplier tube connected to an oscilloscope, and scintillators were excited by a pulsed X‐ray generator (Golden, XRS‐4, pulse width: 15 ns). All PLQY data of QDs were collected using an Edinburgh Instruments FLS 1000 photoluminescence spectrometer. The optical density (OD) values of the QD samples at the excitation wavelength were all in the range of 0.02–0.05. The XRD data were obtained through Empyrean using Cu‐Kα radiation (λ = 1.54 Å). The TEM characterization was conducted using two instruments: a JEOL JEM‐F200 and a Thermo Fisher Scientific‐Titan Themis G2 60–300 microscope equipped with an EDAX energy‐dispersive X‐ray spectroscopy (EDS) detector. Both systems operated at an accelerating voltage of 200 kV during the analyses. The contact angle of a water droplet on the sample surface was conducted using a contact angle tester (SDC 100, Sindin, China) at room temperature. The X‐ray attenuation coefficient data of CdZnS, ZnS, CdS, and CsPbBr_3_ as a function of X‐ray photon energy can be obtained from the XCOM (NIST) database. The X‐ray imaging system was self‐assembled. The light path was deflected by a prism at 90°, and photos were collected by the camera. The prism was used to prevent direct exposure of X‐rays to the camera and minimize potential harm. For the dynamic imaging demonstration, an injection pump was mounted on a scanning stage at an injection rate of 20 mm s^−1^. The camera operated in an automatic acquisition mode.

## Conflict of Interest

The authors declare no conflict of interest.

## Supporting information



Supporting Information

Supplemental Movie 1

## Data Availability

The data that support the findings of this study are available from the corresponding author upon reasonable request.
