# Supplementary material for: Type‐I Heterostructure CdZnS/ZnS Core/Shell Quantum Dots Scintillators for Stable, High‐Resolution, and Real‐Time X‐Ray Imaging
Source: Adv Sci (Weinh). 2025 Oct 6;12(48):e15465. doi: 10.1002/advs.202515465 (PMC12752668; doi:10.1002/advs.202515465)
Supplement: Supplementary file 1 — Supporting Information [file ADVS-12-e15465-s001.docx]

Supporting Information

**Type-Ⅰ Heterostructure CdZnS/ZnS Core/Shell Quantum Dots Scintillators for Stable, High-Resolution, and Real-Time X-Ray Imaging**

Ouyang Wang, Fei Zhang^*^, Rui Zhang, Meng Wang, Wenqing Liang^*^, Zhifeng Shi^*^ and Xinjian Li^*^

Ouyang Wang, Rui Zhang, Meng Wang, Prof. Zhifeng Shi, Prof. Xinjian Li

Key Laboratory of Material Physics of Ministry of Education, School of Physics, Zhengzhou University, Daxue Road 75, Zhengzhou 450052, China

E-mail: shizf@zzu.edu.cn; lixj@zzu.edu.cn
Prof. Fei Zhang, Dr. Wenqing Liang

School of Flexible Electronics (SoFE), Henan Institute of Flexible Electronics (HIFE), Henan University, 379 Mingli Road, Zhengzhou 450046, China

E-mail: ifefzhang@henu.edu.cn; wq_liang@outlook.com

Keywords: CdZnS/ZnS quantum dots scintillators, core/shell structures, stability, real-time X-ray imaging


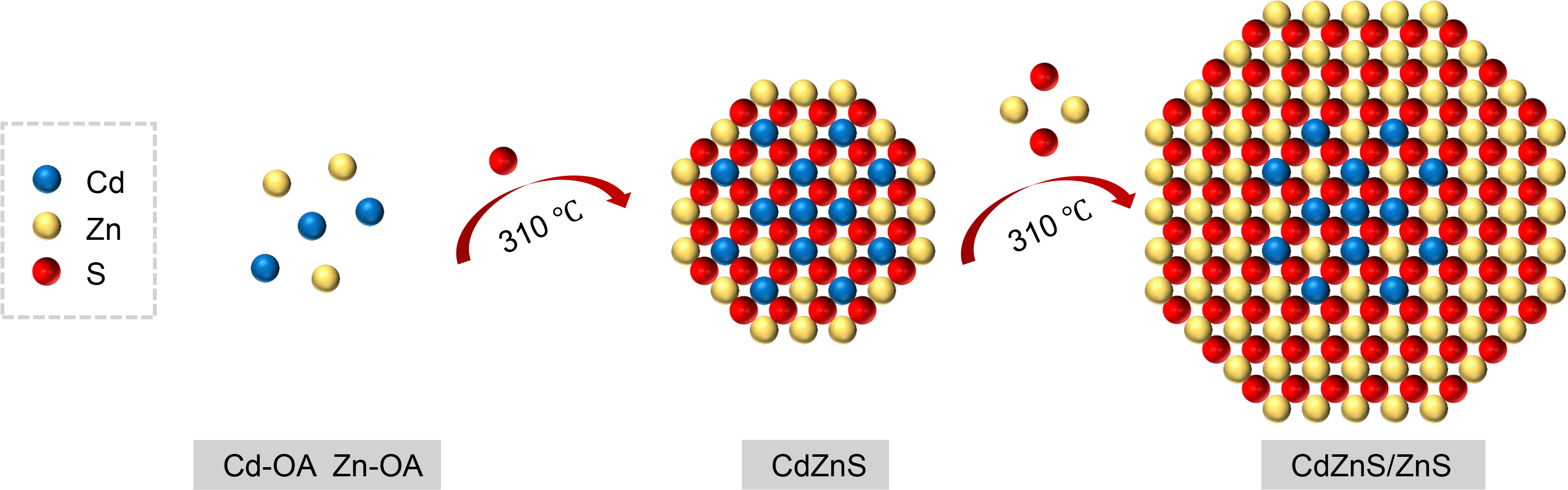


**Figure S1.** Schematic illustration of the synthesis route for Cd_x_Zn_1‒x_ S and Cd_x_Zn_1‒x_S/y ML-ZnS QDs.


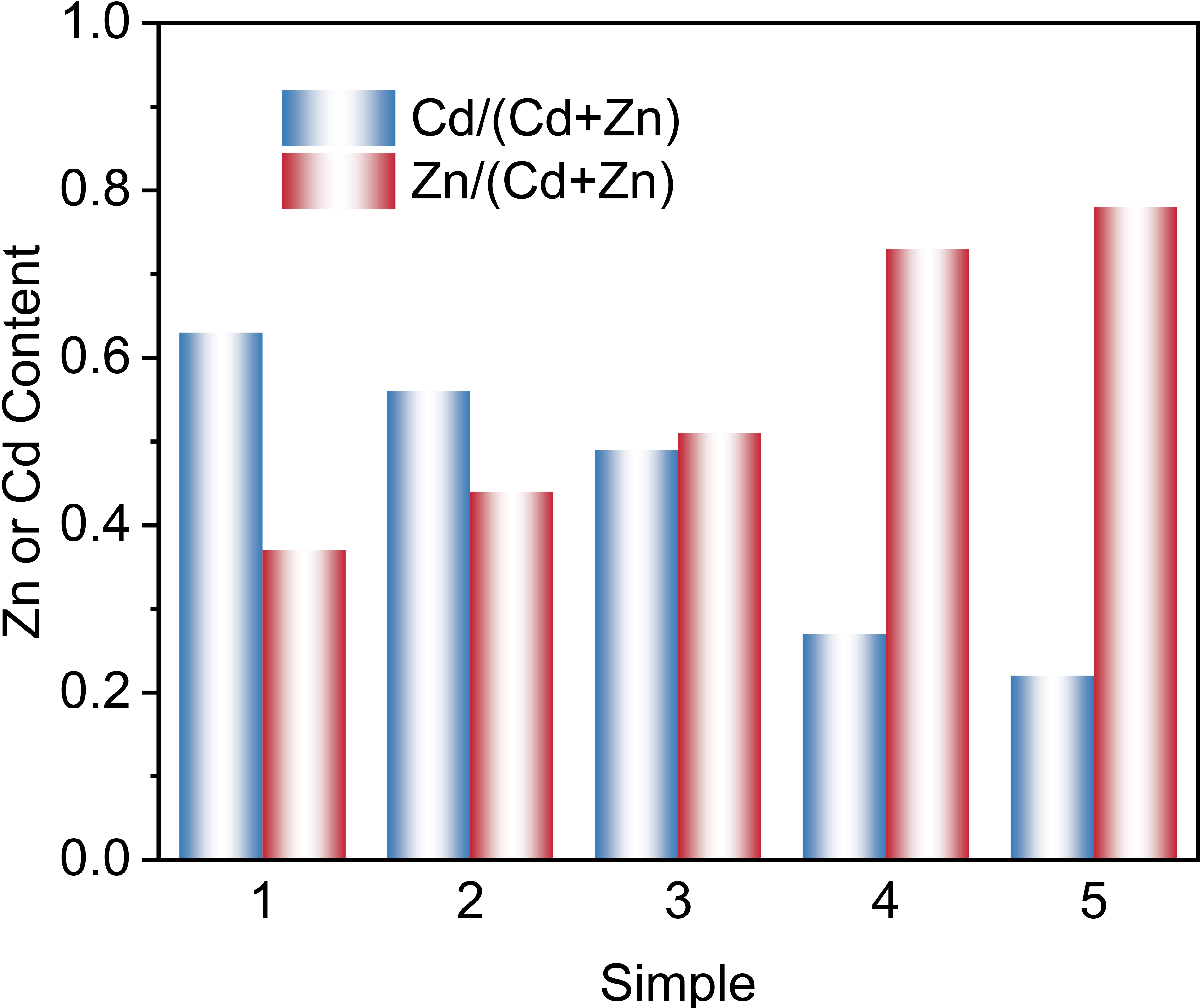


**Figure S2.** The cation ratio within the Cd_1-x_Zn_x_S core QDs in five sample.


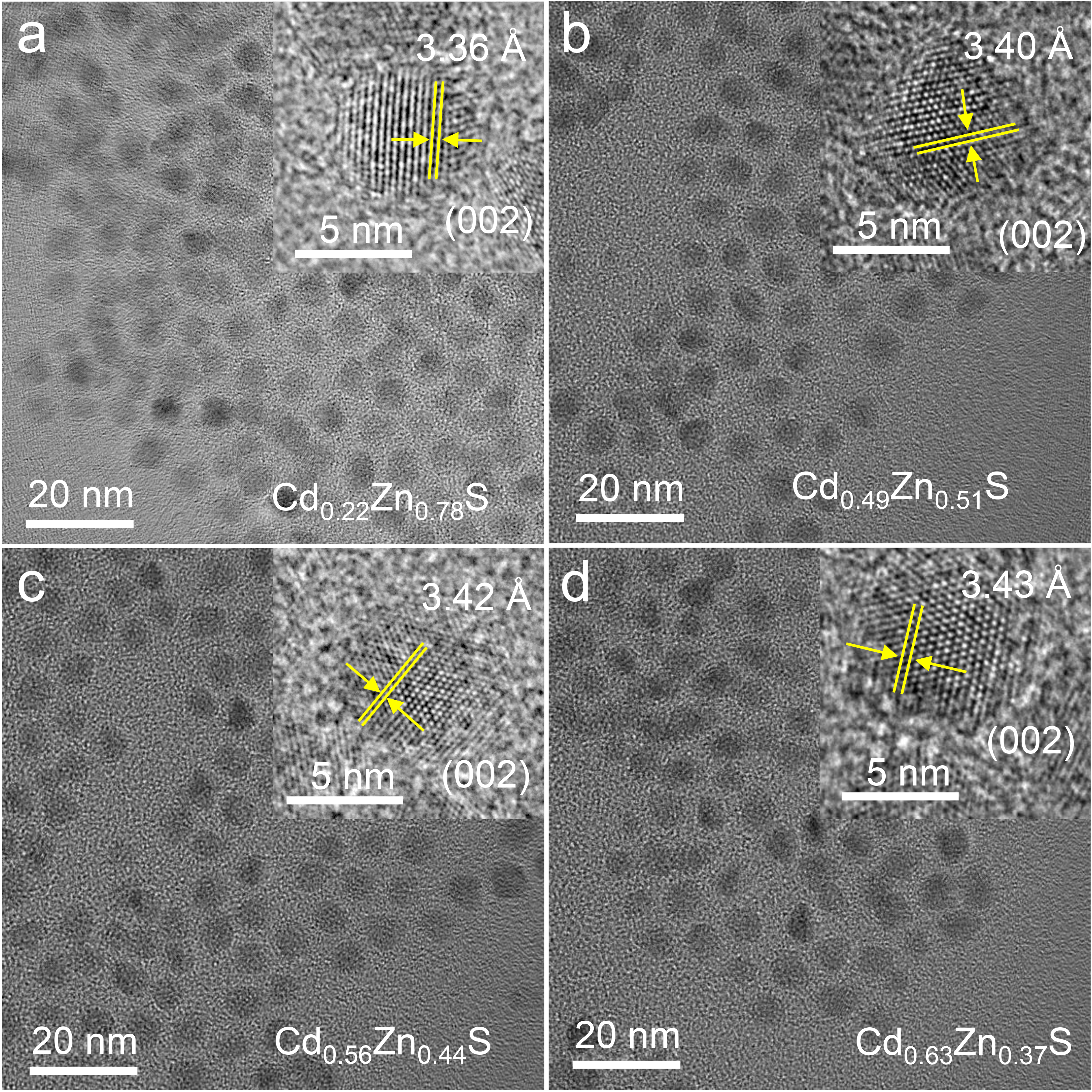


**Figure S3.** TEM image of a) Cd_0.22_Zn_0.78_S QDs, b) Cd_0.49_Zn_0.51_S QDs, c) Cd_0.56_Zn_0.44_S QDs, and d) Cd_0.63_Zn_0.37_S QDs. (Upper right insets are the HRTEM images.)


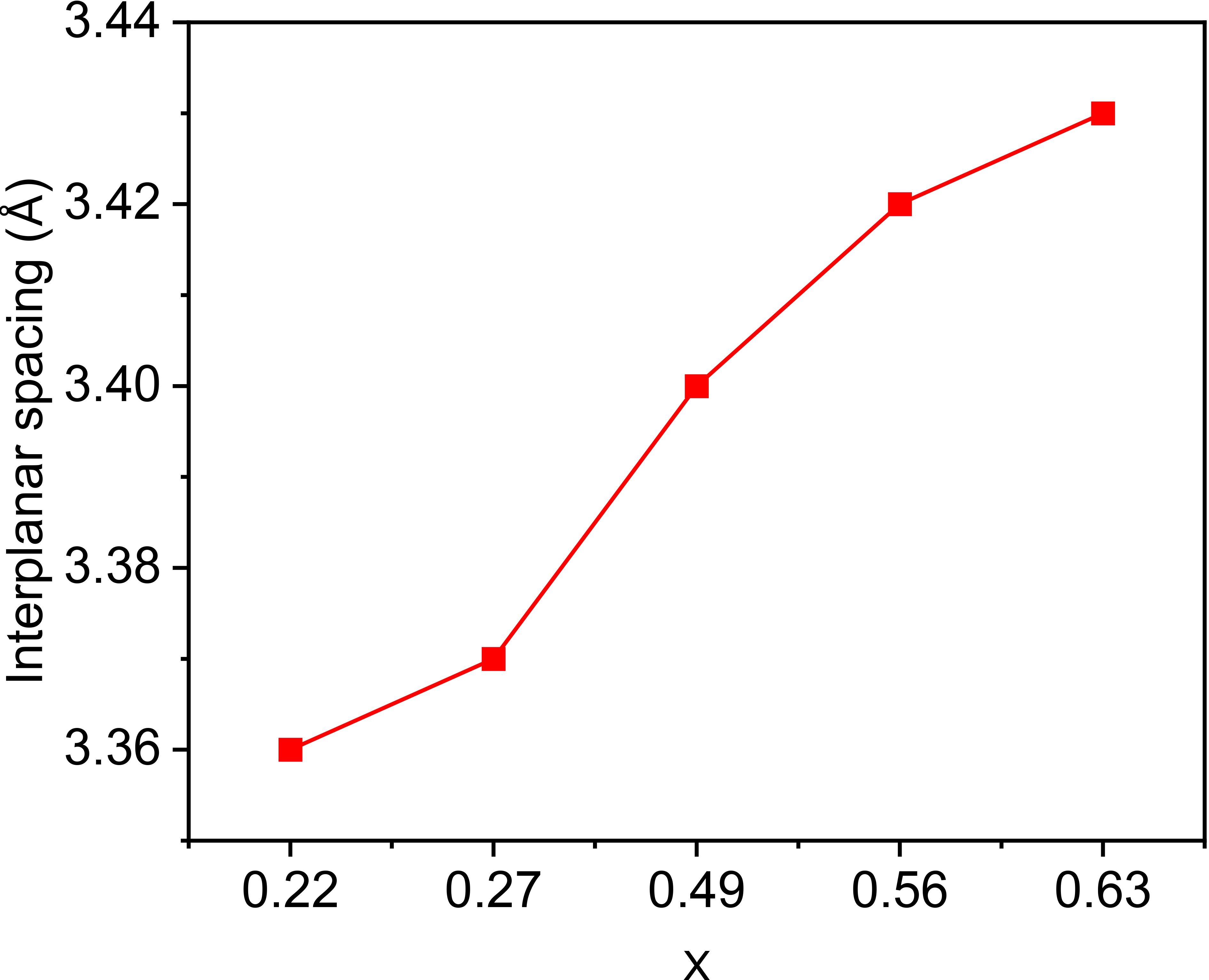


**Figure S4.** The (002) lattice plane spacing of corresponding Cd_x_Zn_1-x_S QDs at different values of x.


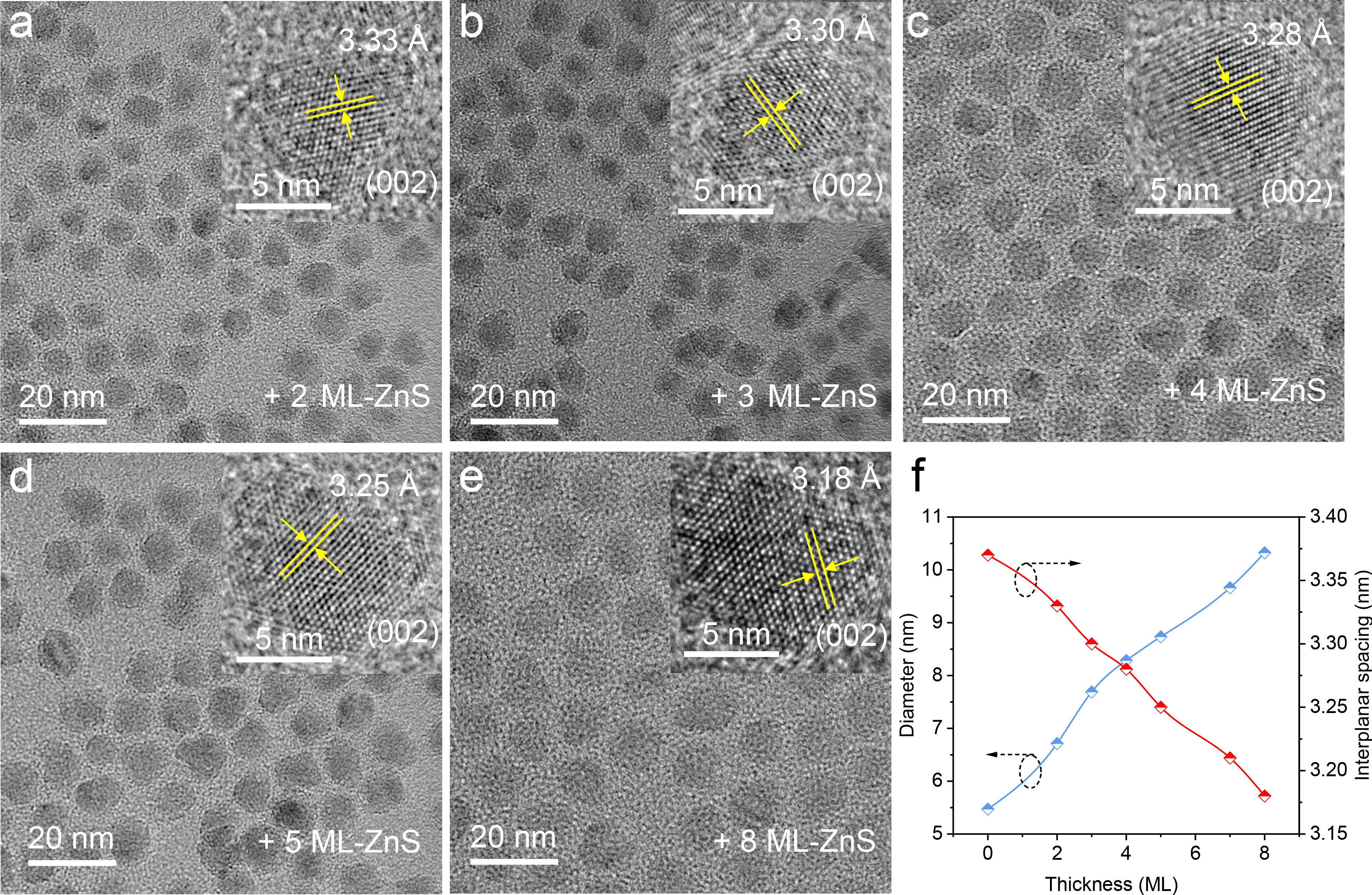


**Figure S5.** TEM image of a) Cd_0.27_Zn_0.73_S/2 ML-ZnS QDs, b) Cd_0.27_Zn_0.73_S/3 ML-ZnS QDs, c) Cd_0.27_Zn_0.73_S/4 ML-ZnS QDs, d) Cd_0.27_Zn_0.73_S/5 ML-ZnS QDs, and e) Cd_0.27_Zn_0.73_S/8 ML-ZnS QDs (Upper right insets are the HRTEM images). f) The (002) lattice plane spacing of corresponding QDs.


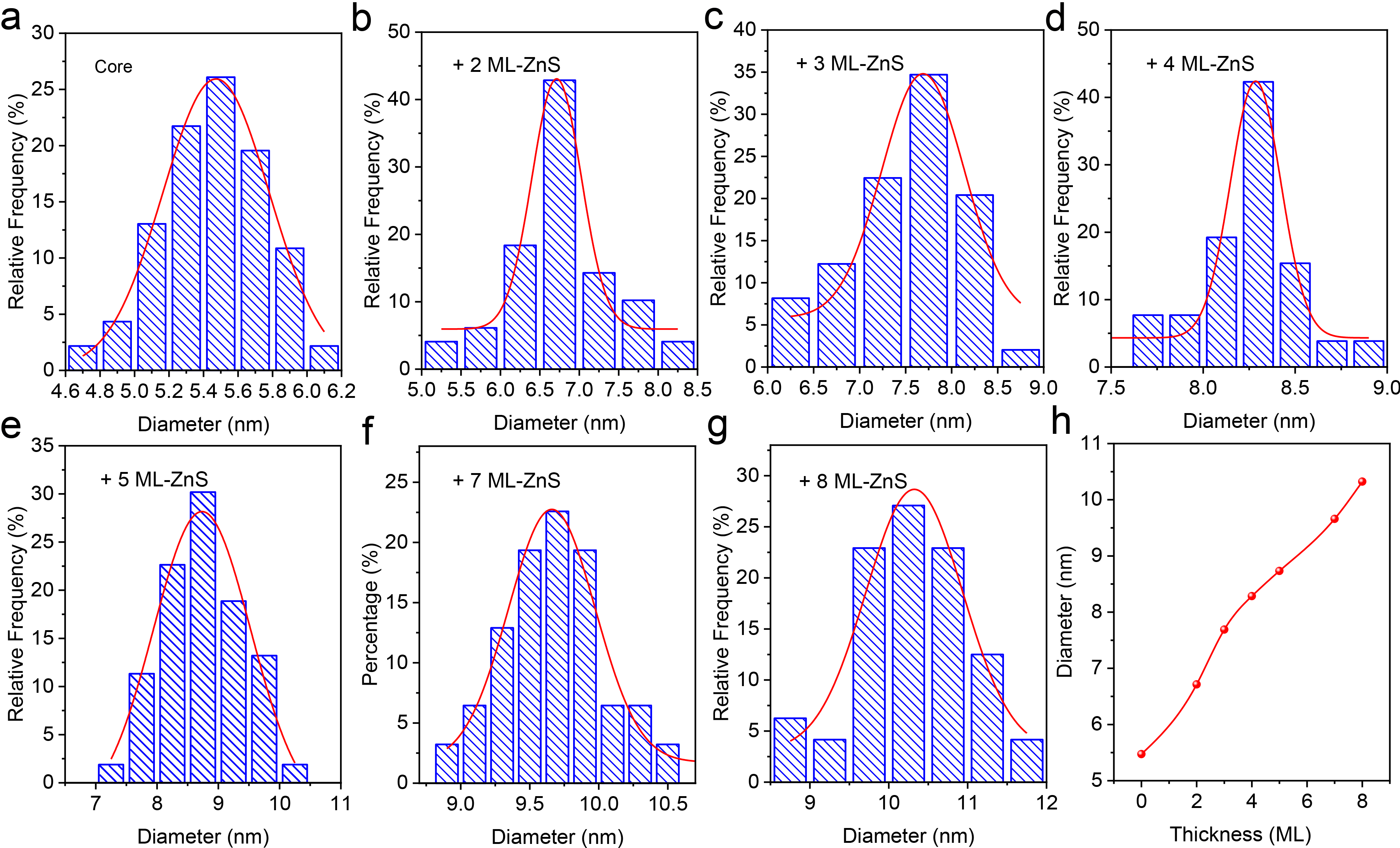


**Figure S6.** Size distribution histogram of a) Cd_0.27_Zn_0.73_S core, b) Cd_0.27_Zn_0.73_S/2 ML-ZnS, c) Cd_0.27_Zn_0.73_S/3 ML-ZnS, d) Cd_0.27_Zn_0.73_S/4 ML-ZnS, e) Cd_0.27_Zn_0.73_S/5 ML-ZnS, f) Cd_0.27_Zn_0.73_S/7 ML-ZnS, and g) Cd_0.27_Zn_0.73_S/8 ML-ZnS, h) variation of diameter with shell thickness.


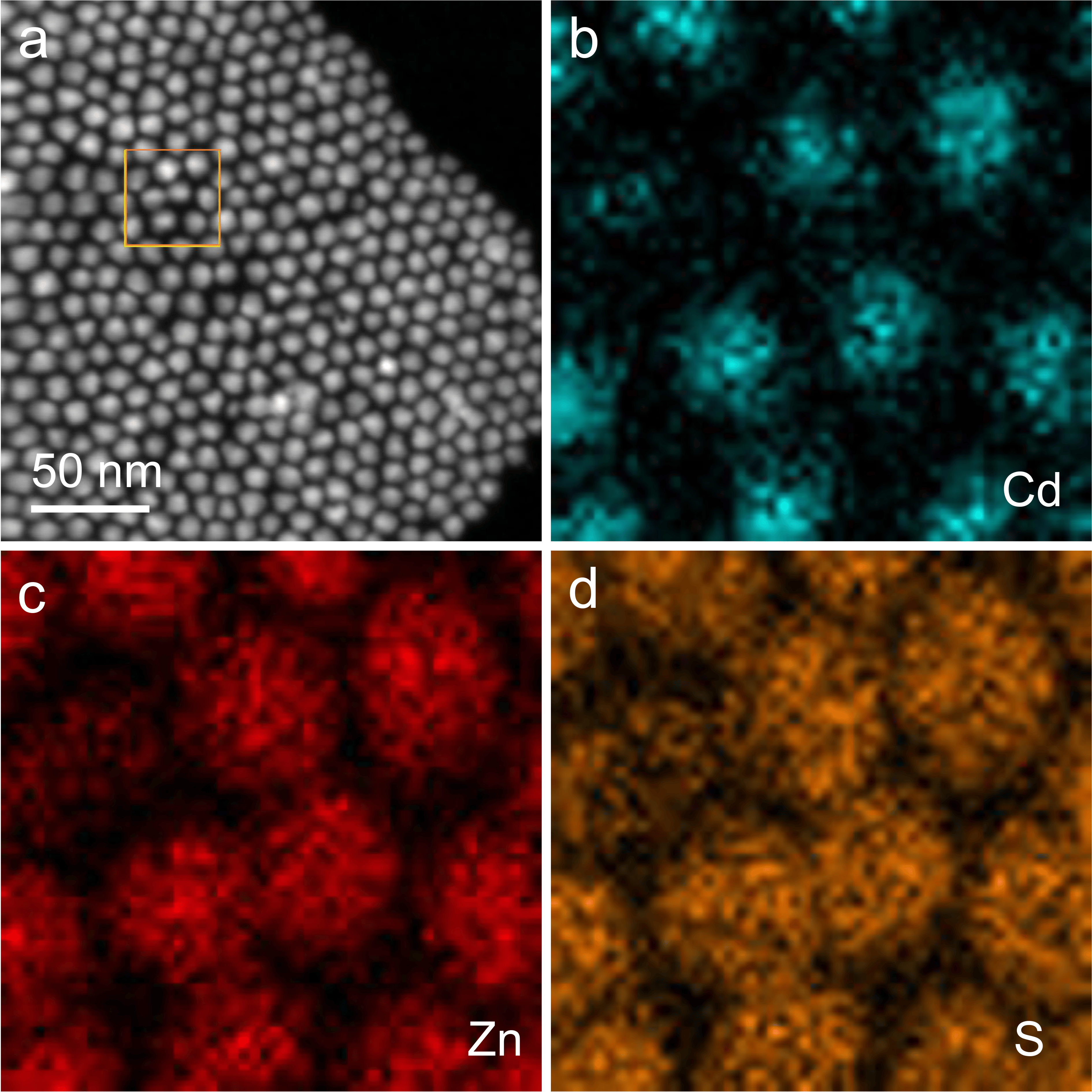


**Figure S7.** TEM images and EDS elemental maps of d Cd_0.27_Zn_0.73_S/7 ML-ZnS core/shell QDs.


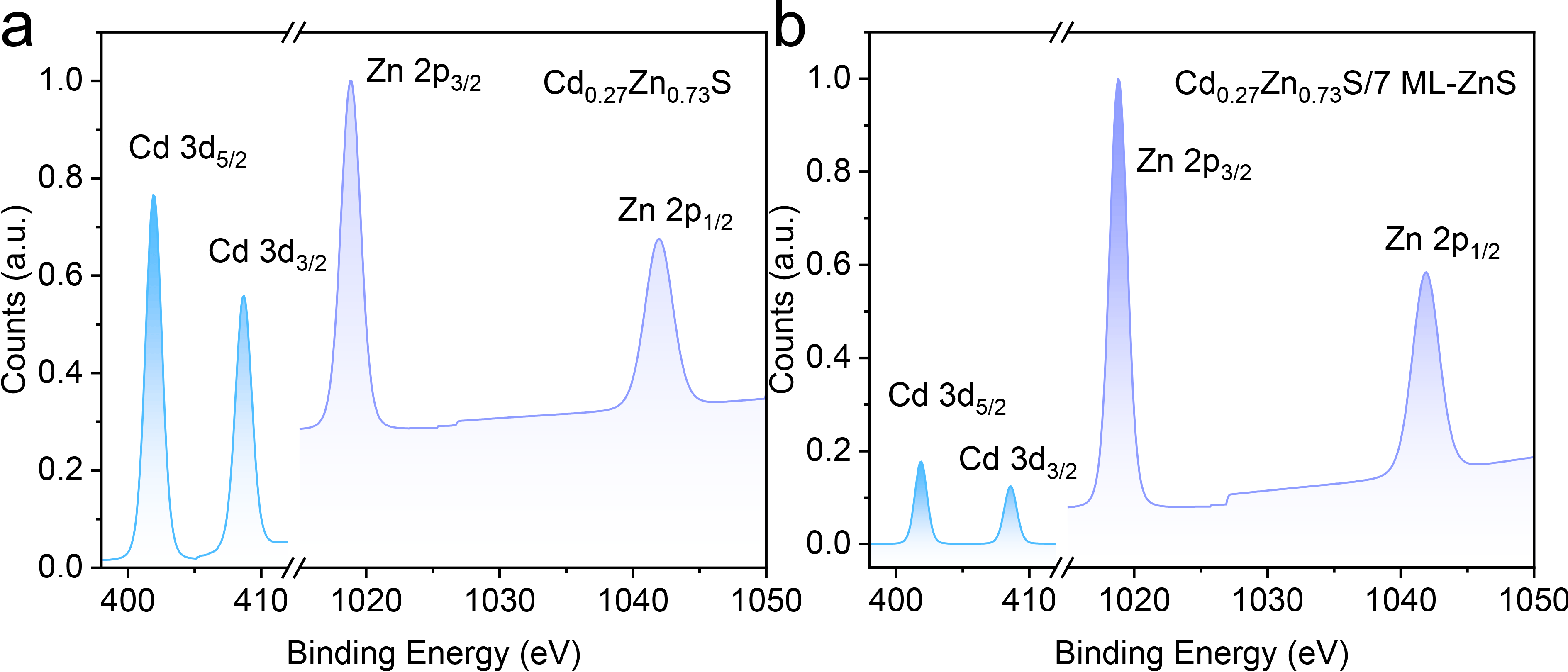


**Figure S8.** High-resolution XPS spectra of Cd 3d and Zn 2p of a) Cd_0.27_Zn_0.73_S core QDs and b) Cd_0.27_Zn_0.73_S/7 ML-ZnS core/shell QDs.


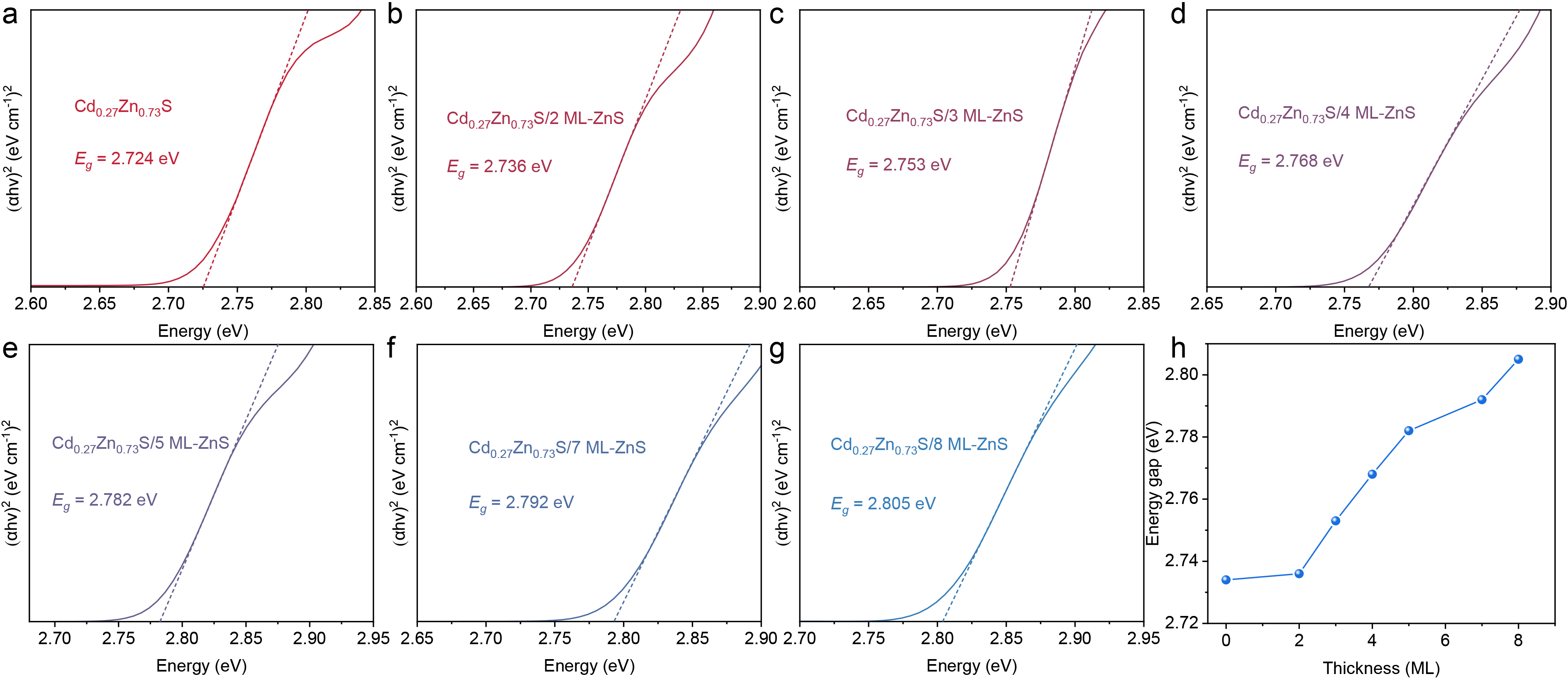


**Figure S9.** The (αhν)^2^-hν relationship curve of a) Cd_0.27_Zn_0.73_S core, b) Cd_0.27_Zn_0.73_S/2 ML-ZnS QDs, c) Cd_0.27_Zn_0.73_S/3 ML-ZnS QDs, d) Cd_0.27_Zn_0.73_S/ 4 ML-ZnS QDs, e) Cd_0.27_Zn_0.73_S/5 ML-ZnS QDs, f) Cd_0.27_Zn_0.73_S/7 ML-ZnS QDs, and g) Cd_0.27_Zn_0.73_S/8 ML-ZnS QDs. h) The shell-thickness dependence of the energy gap of QDs.


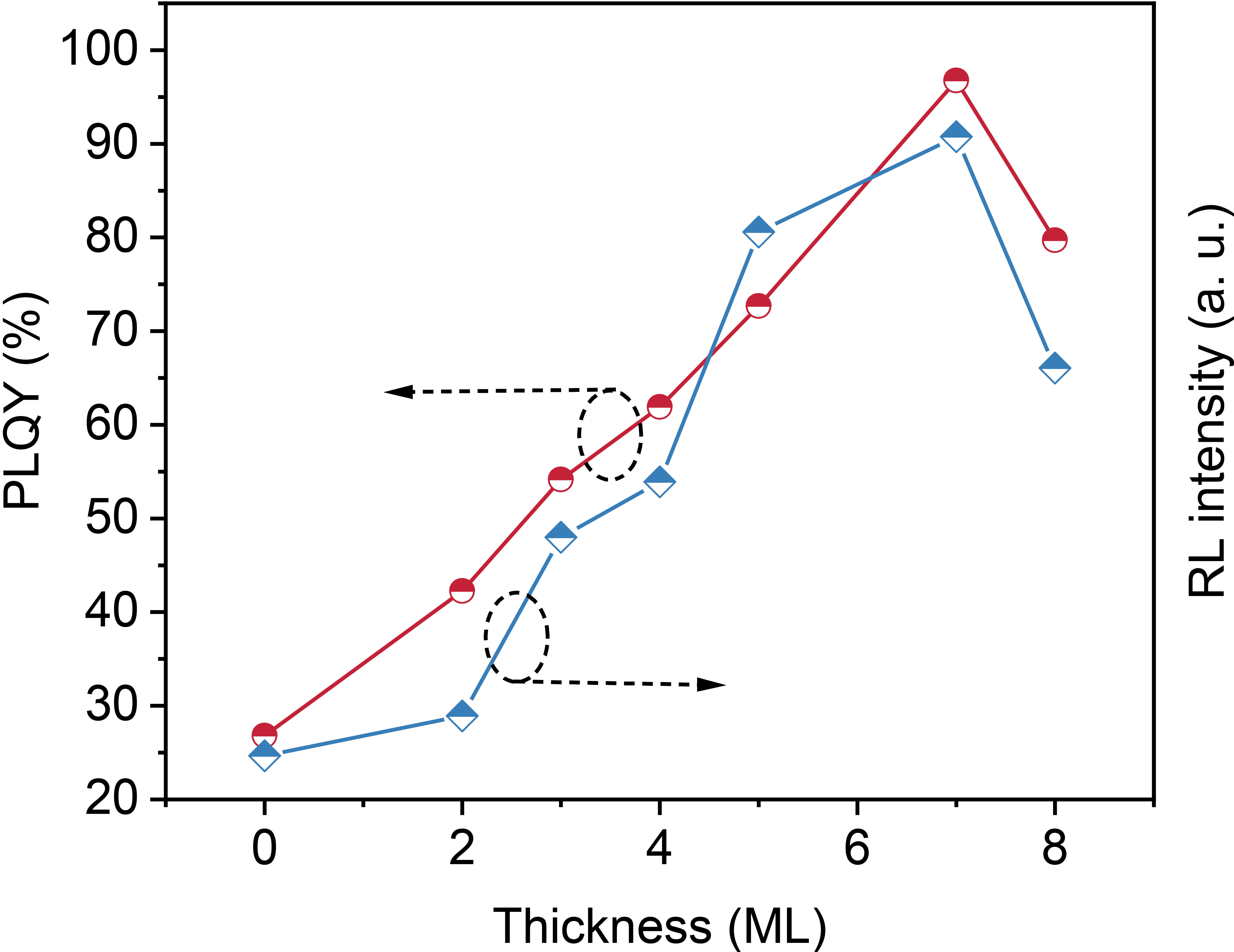


**Figure S10.** PLQY of the Cd_0.27_Zn_0.73_S core and Cd_0.27_Zn_0.73_S/y ML-ZnS QDs with a given number of monolayers (MLs) of the ZnS shell.


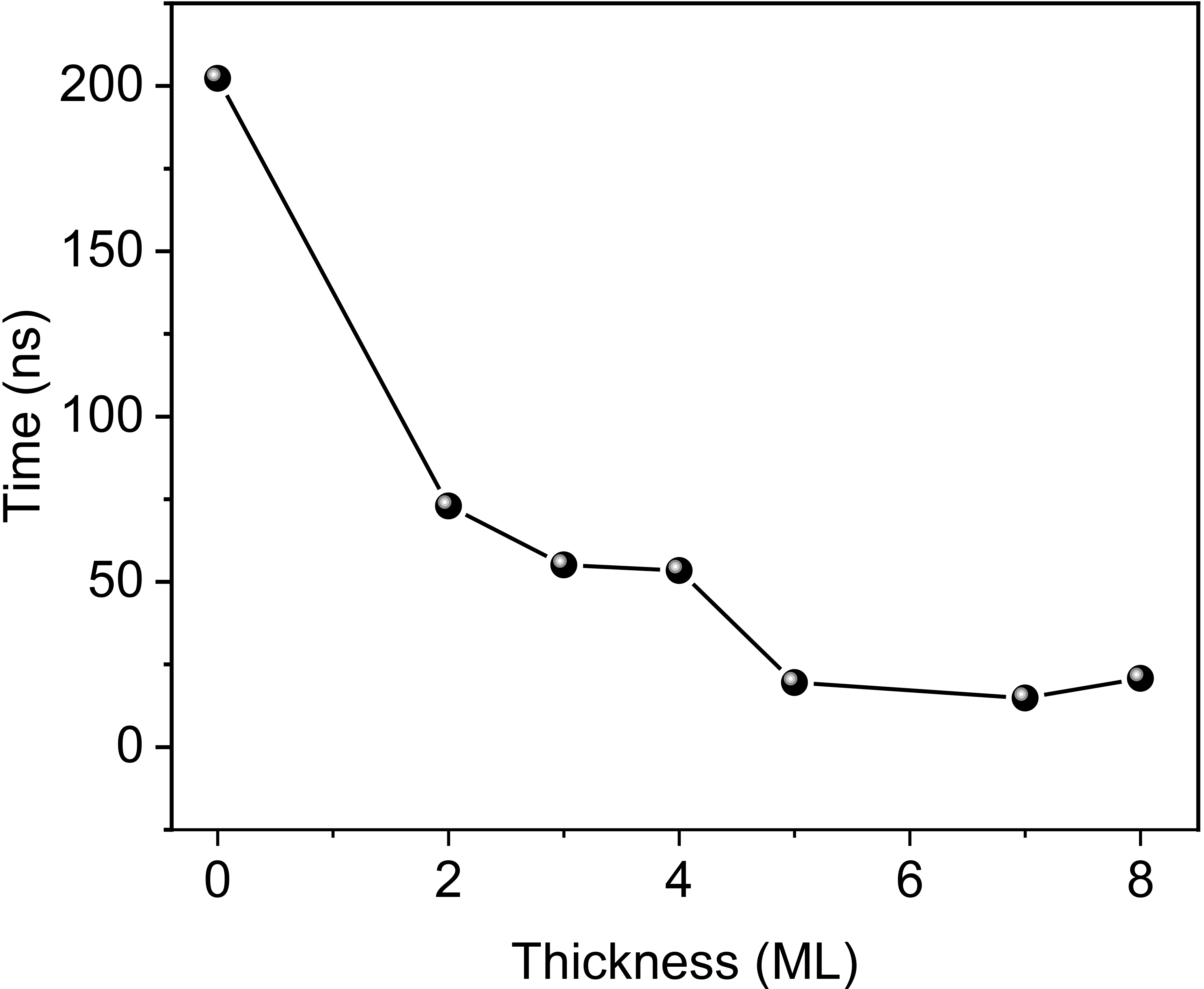


**Figure S11.** The shell-thickness dependence of the average PL decay lifetime of QDs.


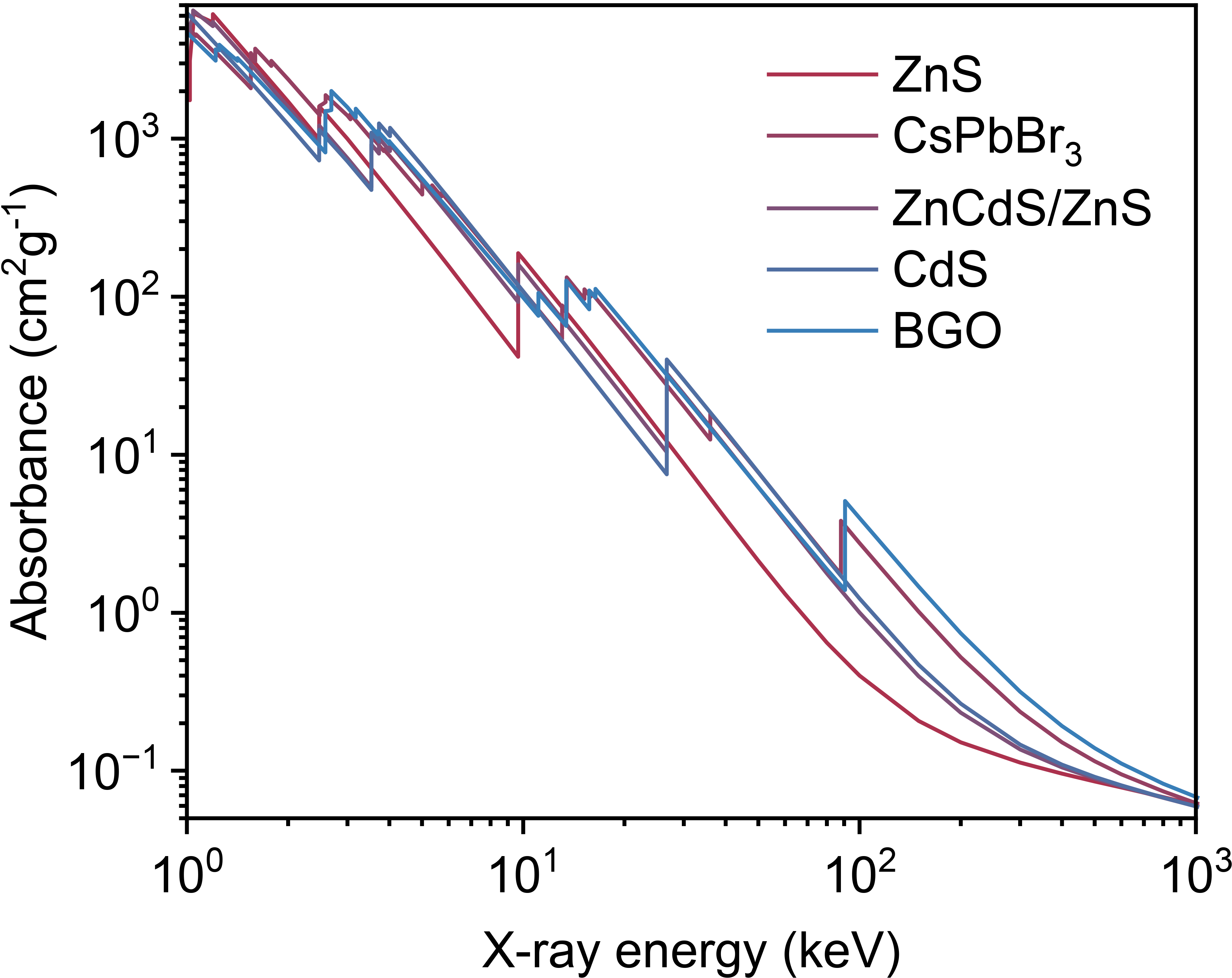


**Figure S12.** The X-ray attenuation coefficient diagram of ZnS, CsPbBr_3_, Cd_0.27_Zn_0.73_S/7 ML-ZnS, CdS and BGO acquired from the NIST XCOM database.


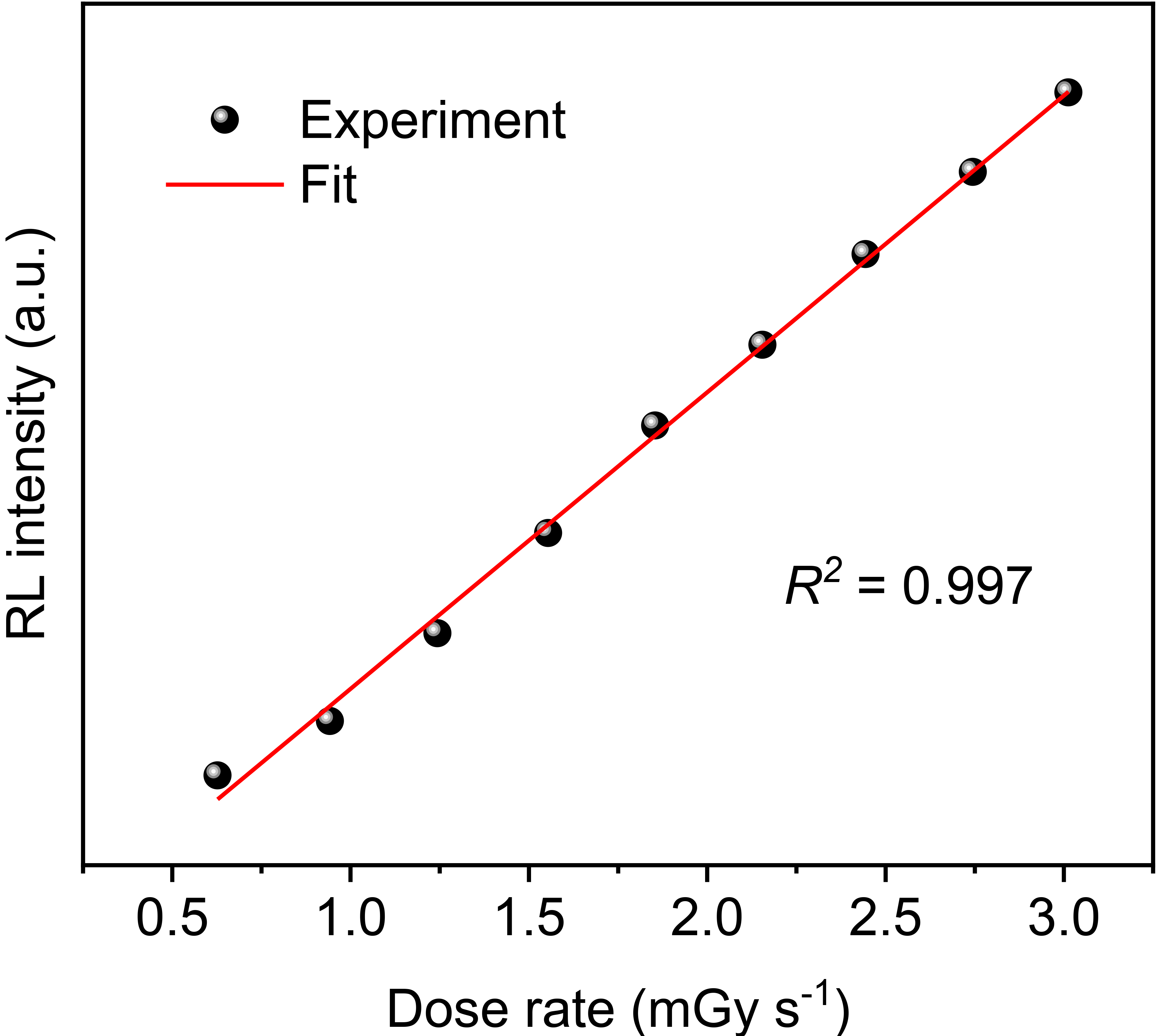


**Figure S13.** The recorded RL intensity of Cd_0.27_Zn_0.73_S/7 ML-ZnS core/shell QDs films as a function of dose rate.


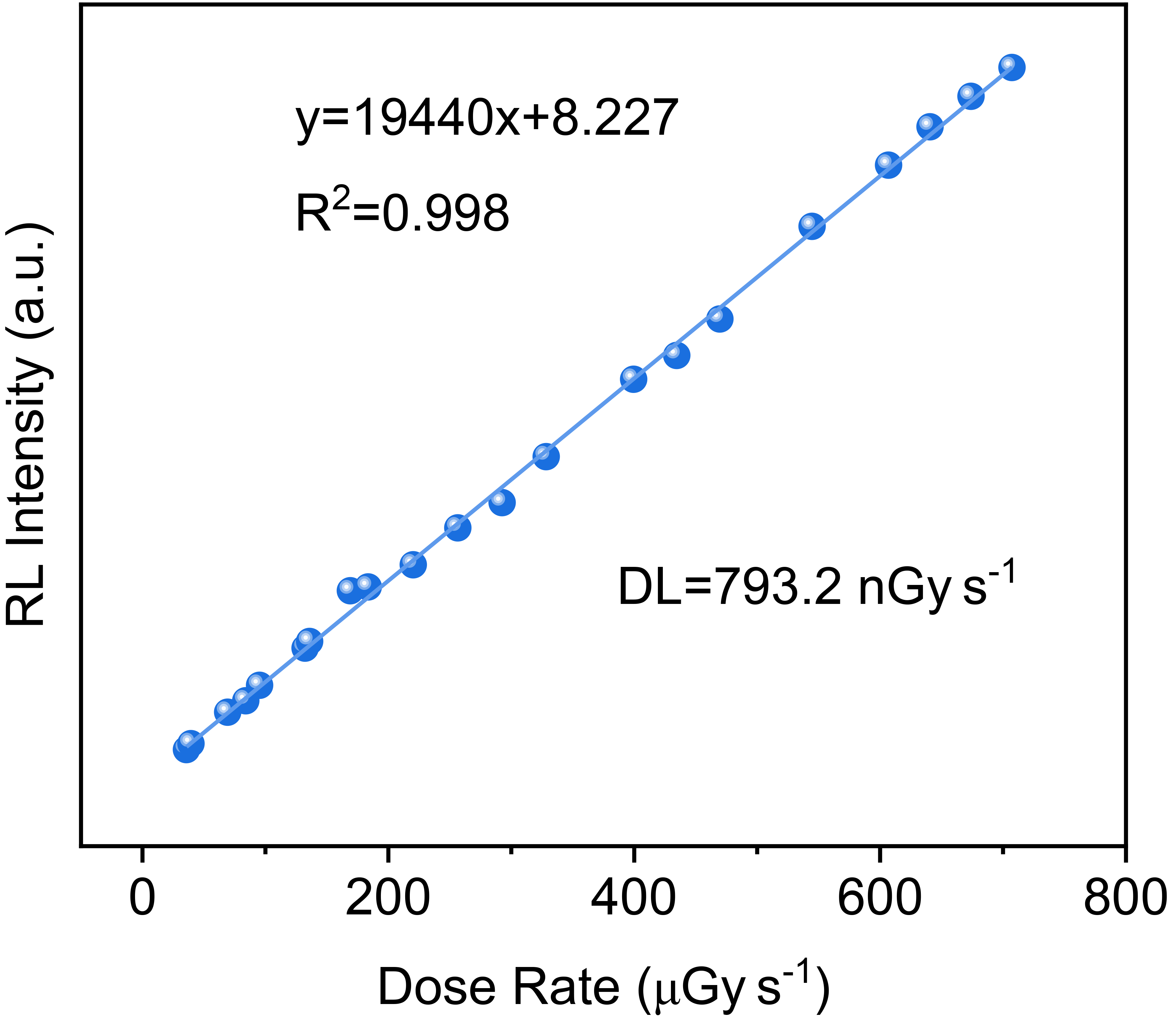


**Figure S14.** The recorded RL intensity of Cd_0.27_Zn_0.73_S/7 ML-ZnS core/shell QDs films as a function of lower dose rate.


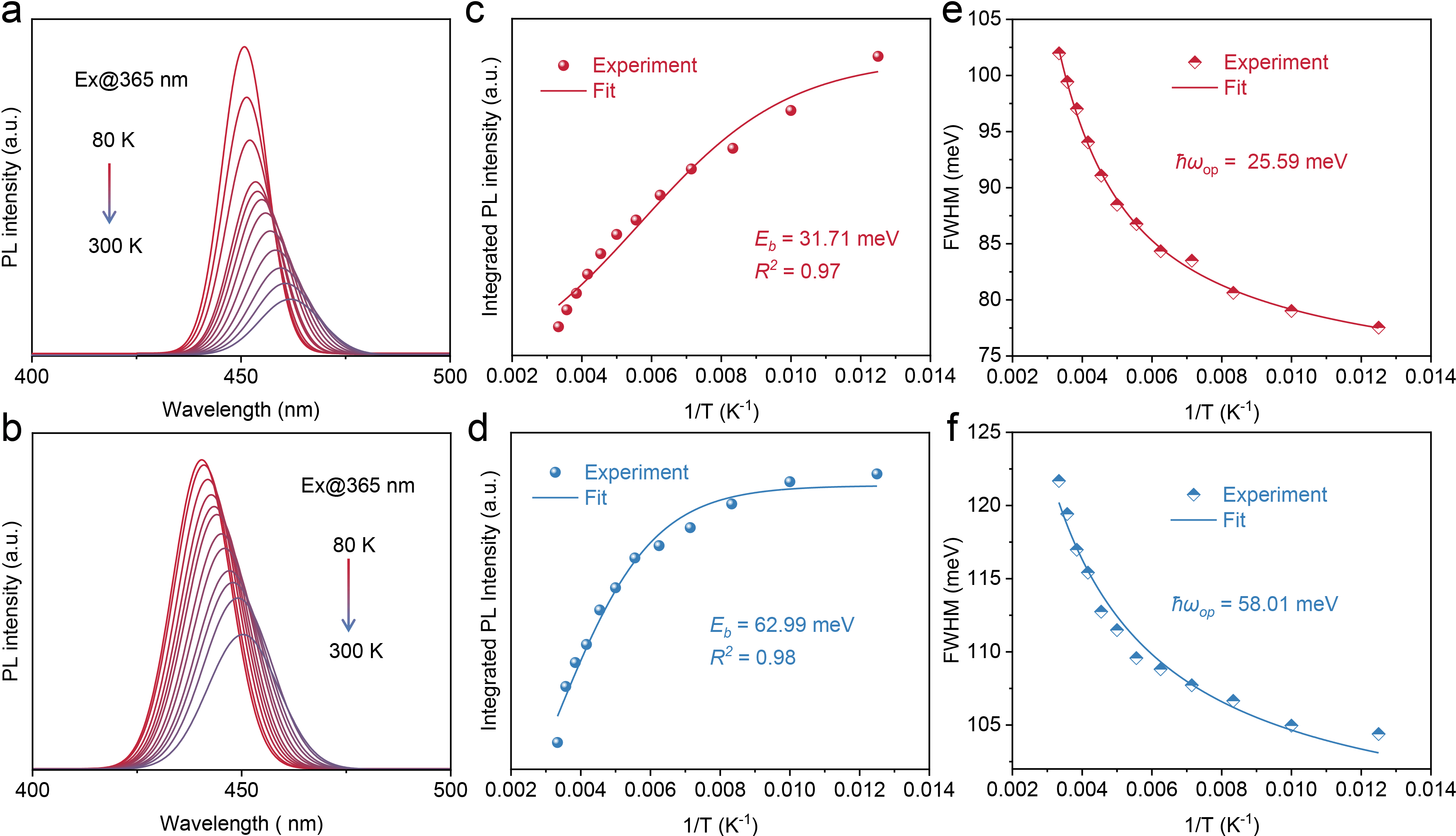


**Figure S15.** Temperature-dependent PL spectra of a) the Cd_0.27_Zn_0.73_S core QDs and b) Cd_0.27_Zn_0.73_S/7 ML-ZnS core/shell QDs taken from 80 to 300 K. The relationship between the integrated PL intensity and the reciprocal temperature from 80 to 300 K for c) Cd_0.27_Zn_0.73_S core QDs and d) Cd_0.27_Zn_0.73_S/7 ML-ZnS core/shell QDs. FWHM of e) Cd_0.27_Zn_0.73_S core QDs and f) Cd_0.27_Zn_0.73_S/7 ML-ZnS core/shell QDs as a function of reciprocal temperature.


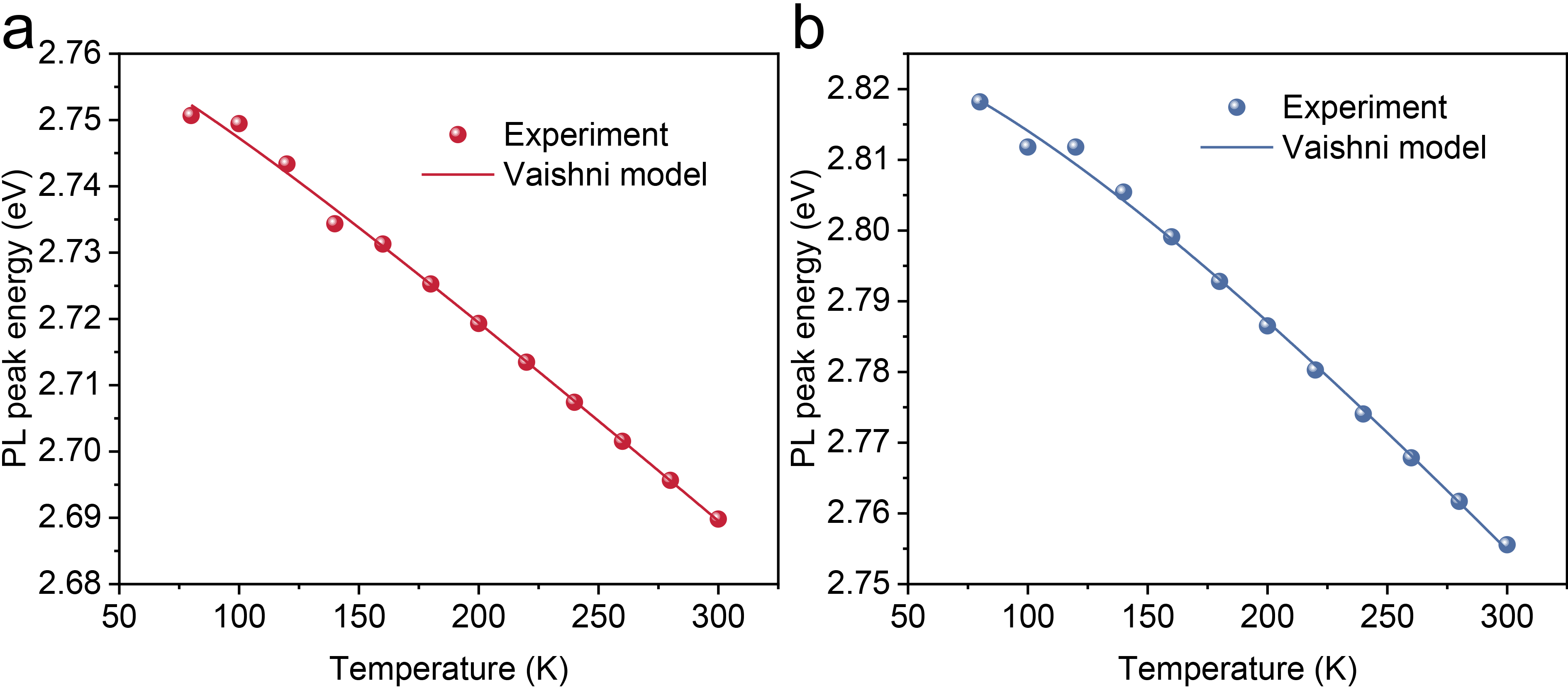


**Figure S16.** PL peak energy of a) the Cd_0.27_Zn_0.73_S core QDs and b) Cd_0.27_Zn_0.63_S/7 ML-ZnS core/shell QDs as a function of the temperature. The lines are the best-fit curves as discussed in the text.


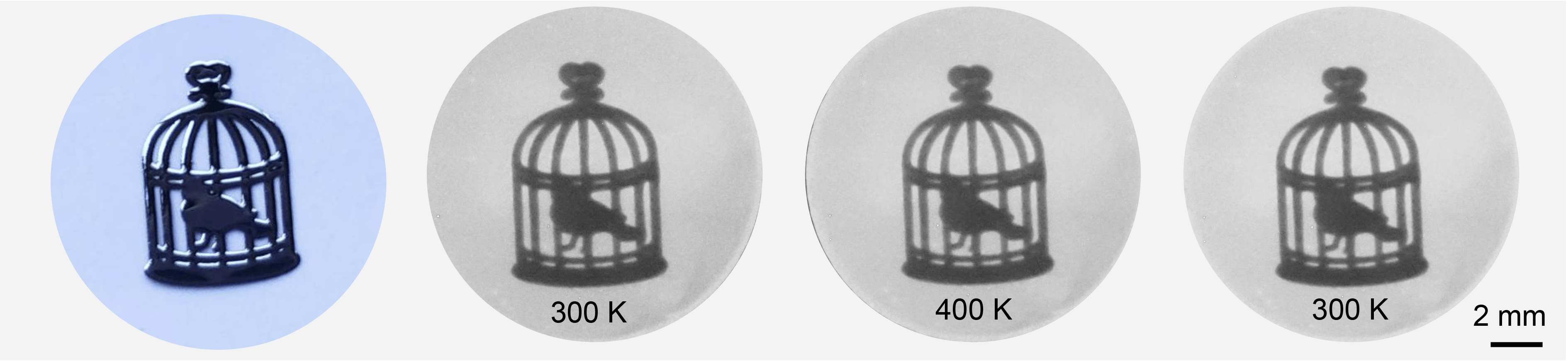


**Figure S17.** Bright-field (left) and X-ray (right) images of the birdcage based on the CdZnS/ZnS @AAO film at 300, 440, and 300 K, respectively.


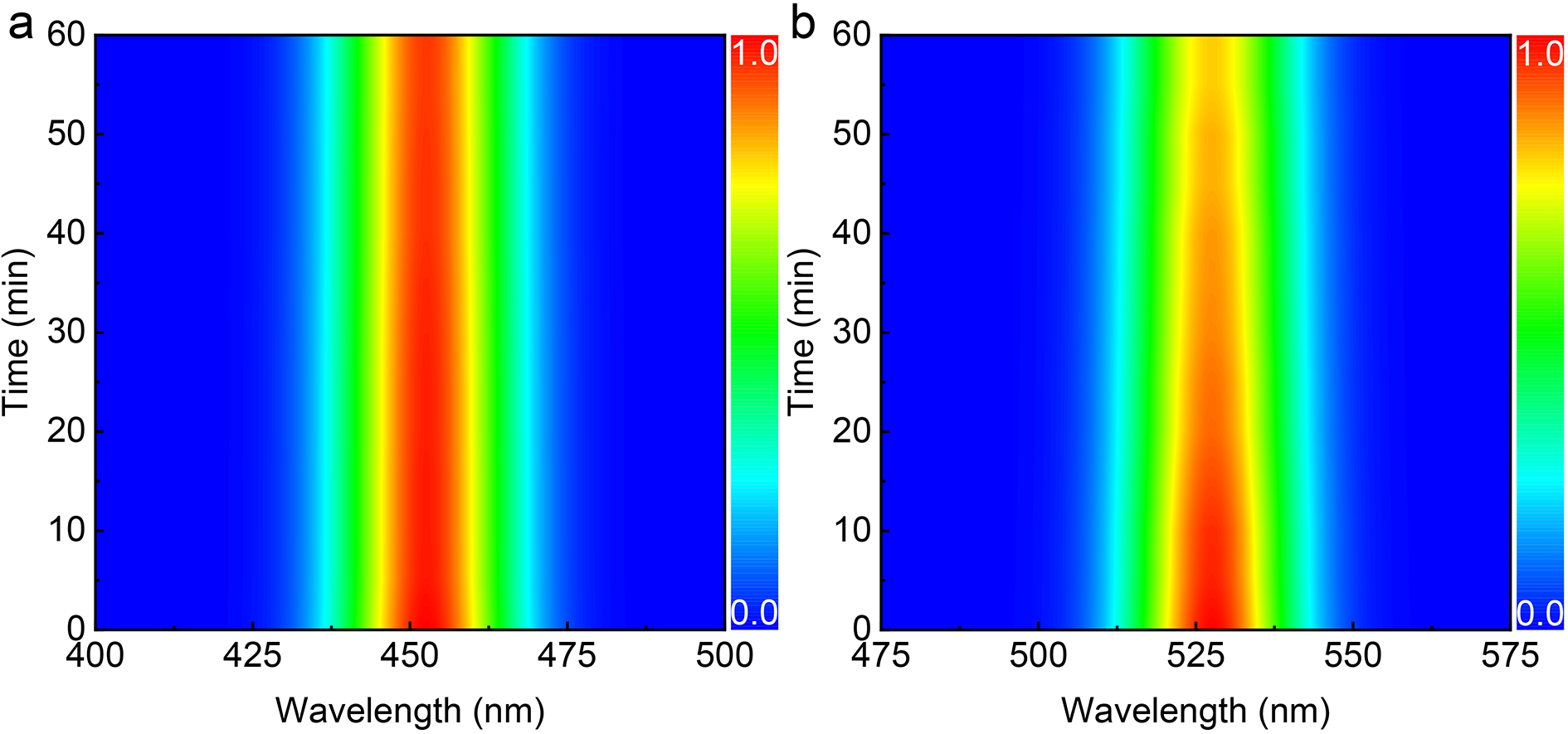


**Figure S18**. Normalized PL spectra of a) Cd_0.27_Zn_0.73_S/7 ML-ZnS core/shell QDs and b) CsPbBr_3_ films under continuous X-ray irradiation.


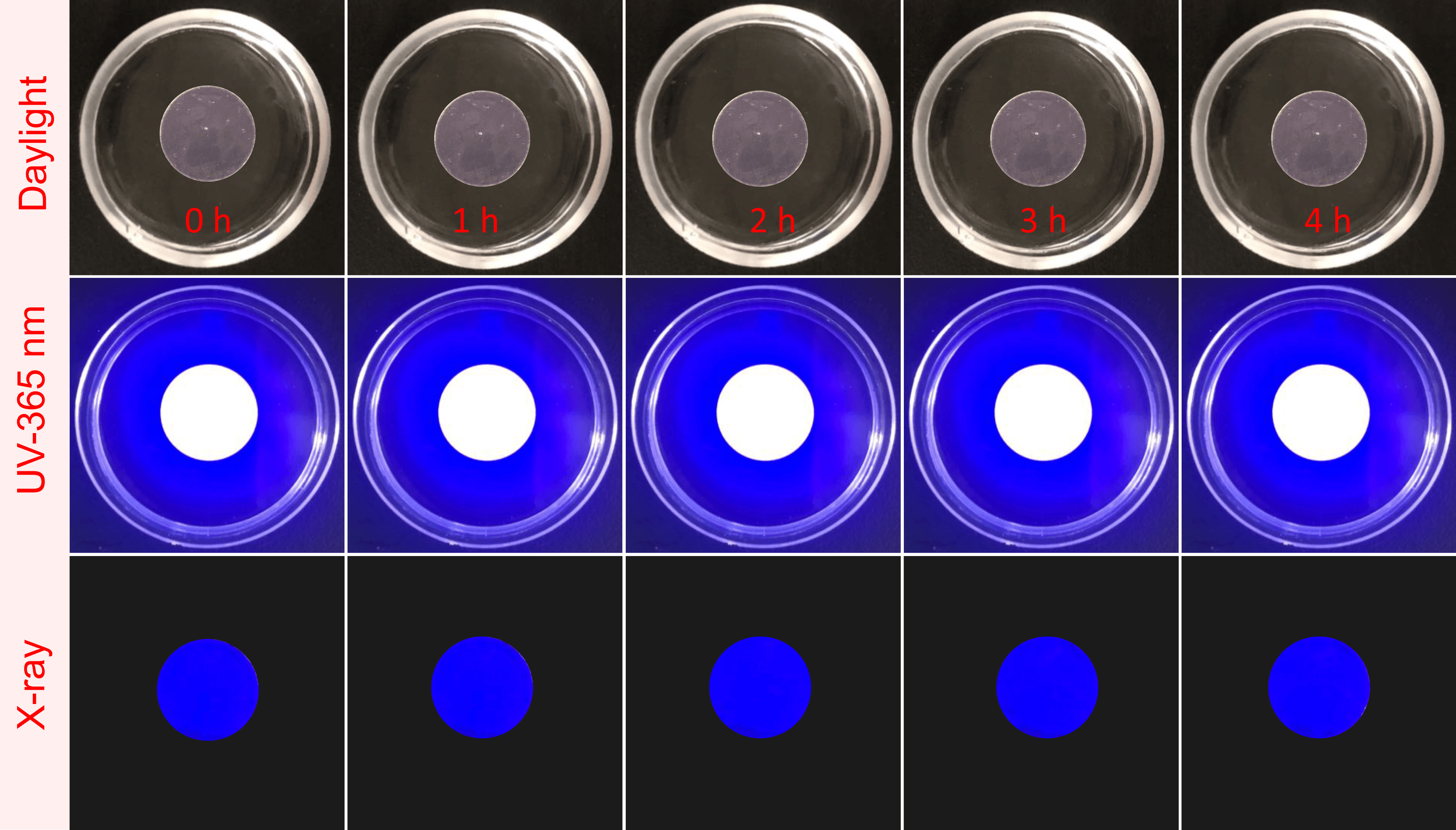


**Figure S19.** Photographs of the Cd_0.27_Zn_0.73_S/7 ML-ZnS QDs film immersed in water for different times under daylight, UV lamp, and X-ray irradiation, respectively.


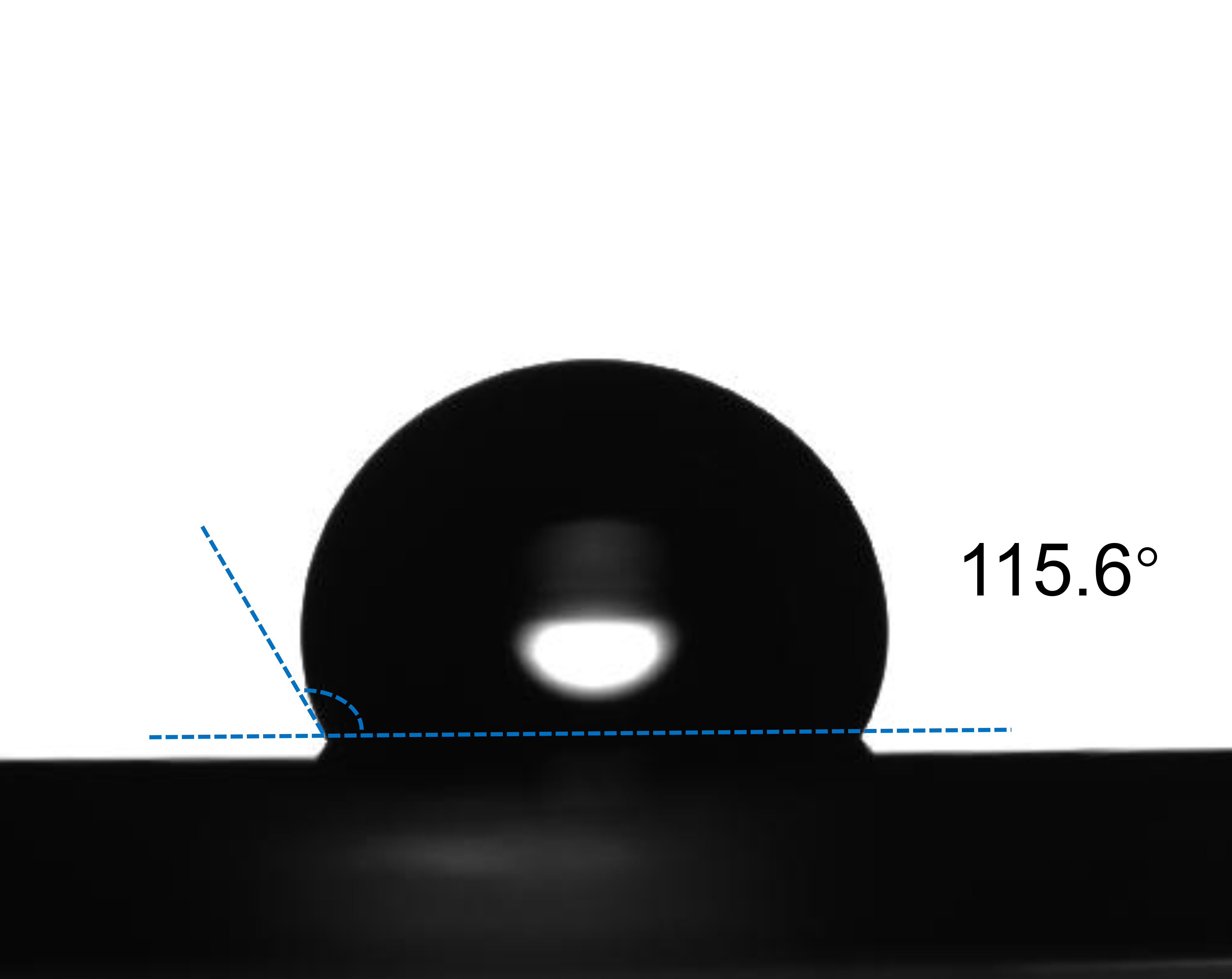


**Figure S20.** The water contact angle of the Cd_0.27_Zn_0.73_S/7 ML-ZnS QDs film.


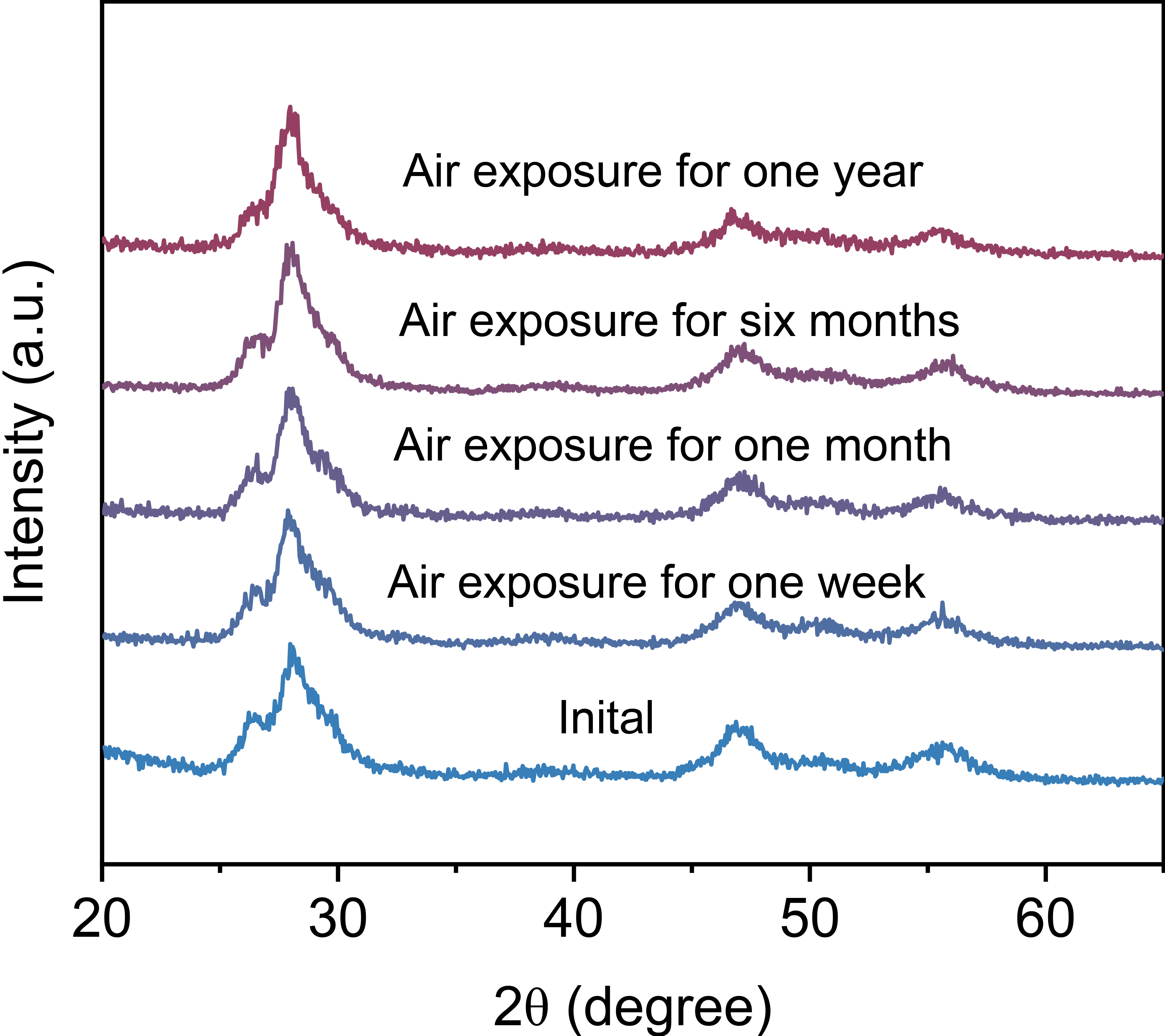


**Figure S21.** The XRD patterns of Cd_0.27_Zn_0.73_S/7 ML-ZnS core/shell QDs exposed to air at different periods.


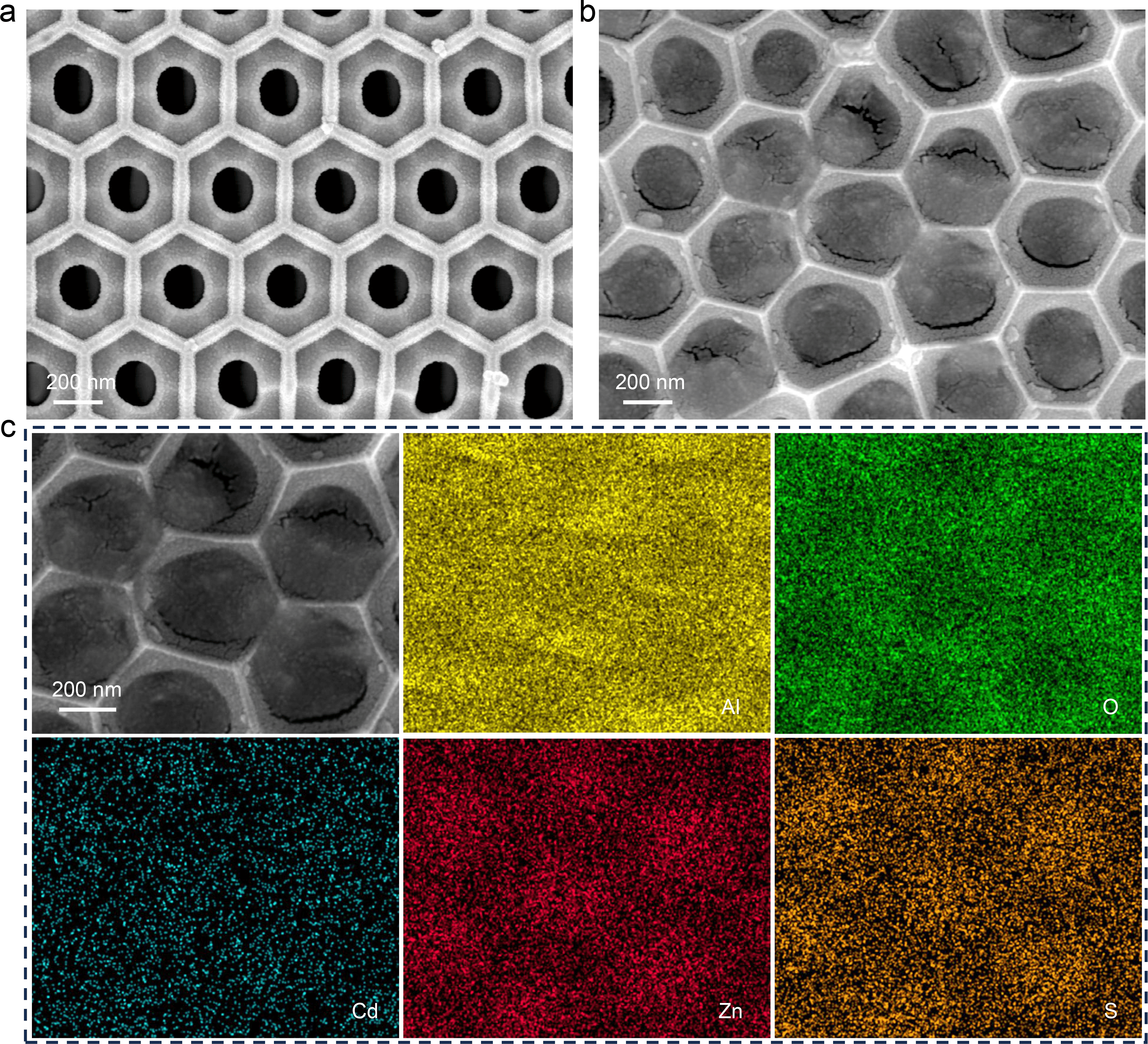


**Figure S22.** Top-view SEM image of a) empty AAO template and b) Cd_0.27_Zn_0.73_S/7 ML-ZnS QDs@AAO, respectively. c) EDS elemental mapping images of Cd_0.27_Zn_0.73_S/7 ML-ZnS QDs@AAO.


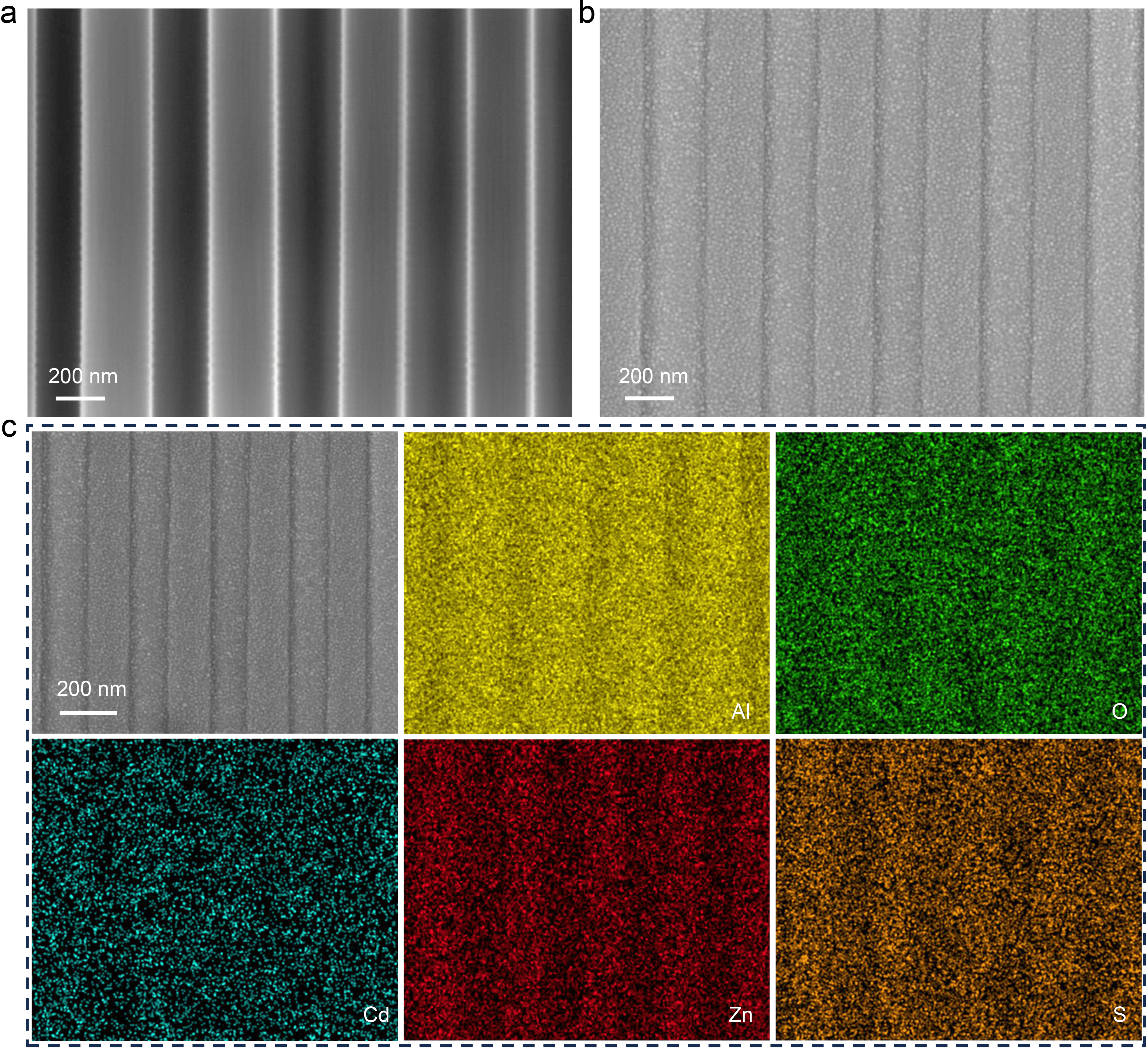


**Figure S23.** Cross-sectional SEM image of a) empty AAO template and b) Cd_0.27_Zn_0.73_S/7 ML-ZnS QDs @AAO arrays. (scale bar: 200 nm), respectively. c) EDS elemental mapping images of Cd_0.27_Zn_0.73_S/7 ML-ZnS QDs@AAO.


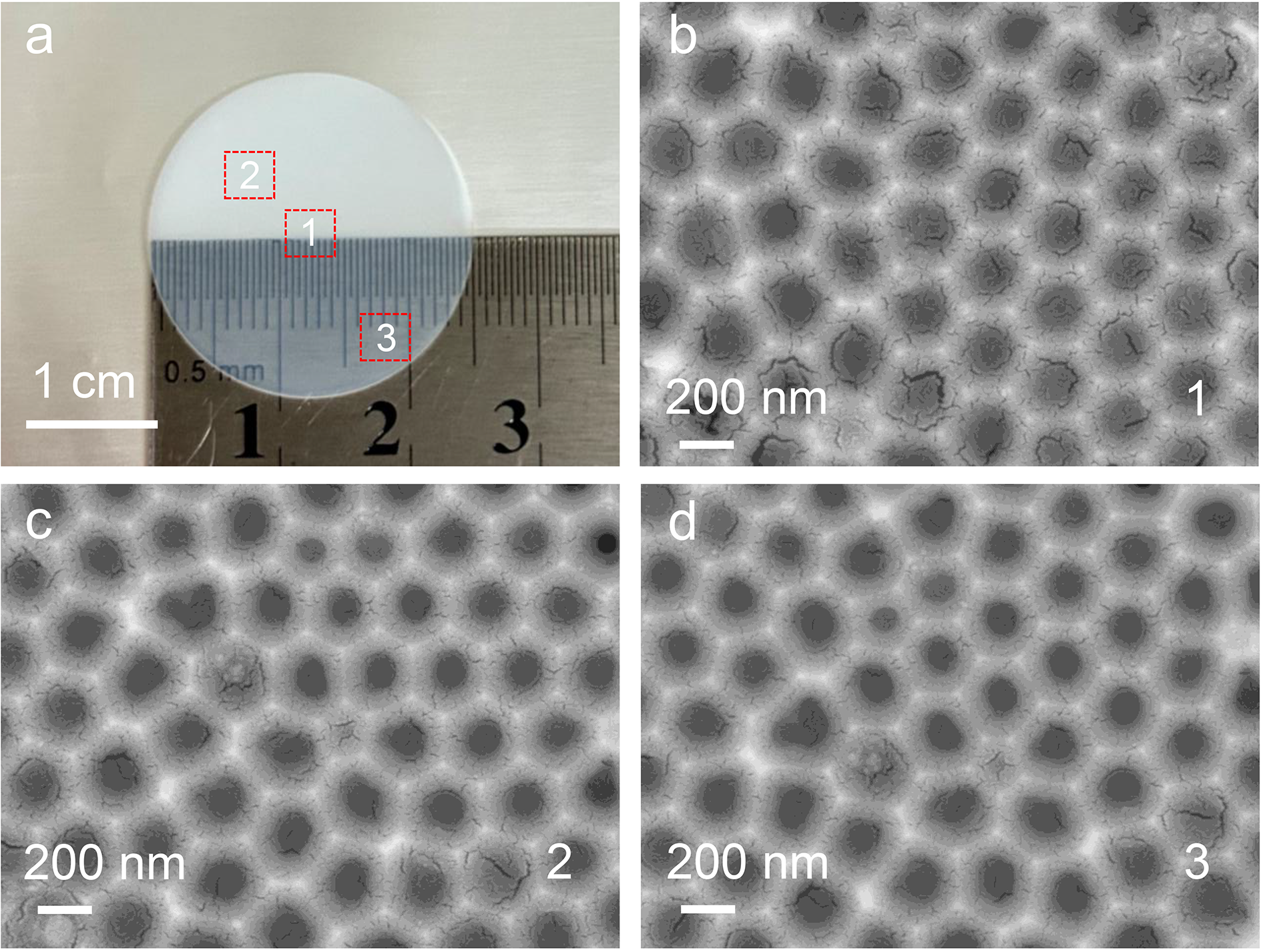


**Figure S24.** a) Photograph of Cd_0.27_Zn_0.73_S/7 ML-ZnS QDs@AAO films with a size of 25 mm (scale bar: 1 cm). Top-view SEM images of the b) center, c) middle, and d) edge regions selected from the sample.


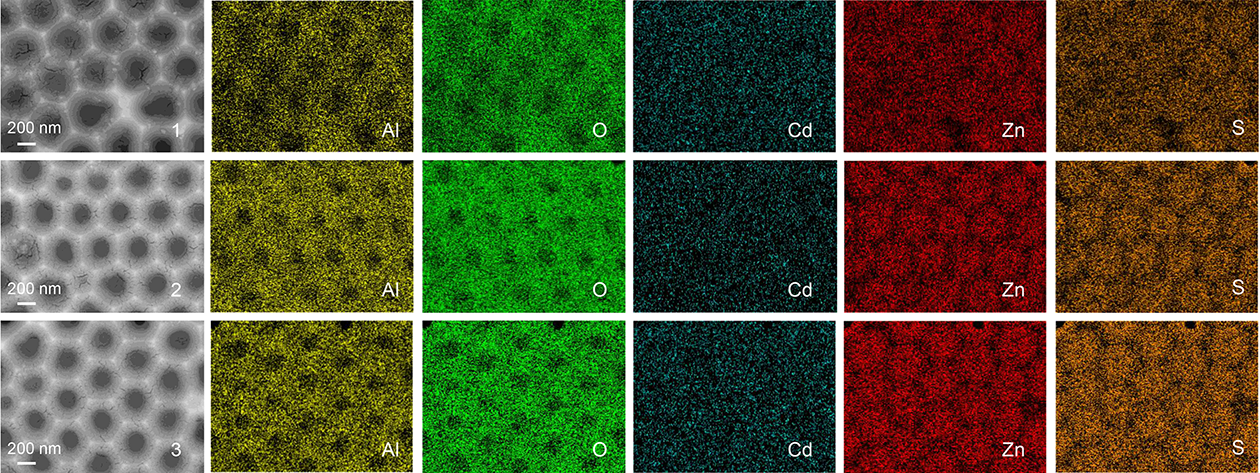


**Figure S25.** EDS elemental mapping images of three selected regions in the Cd_0.27_Zn_0.73_S/7 ML-ZnS QDs@AAO.


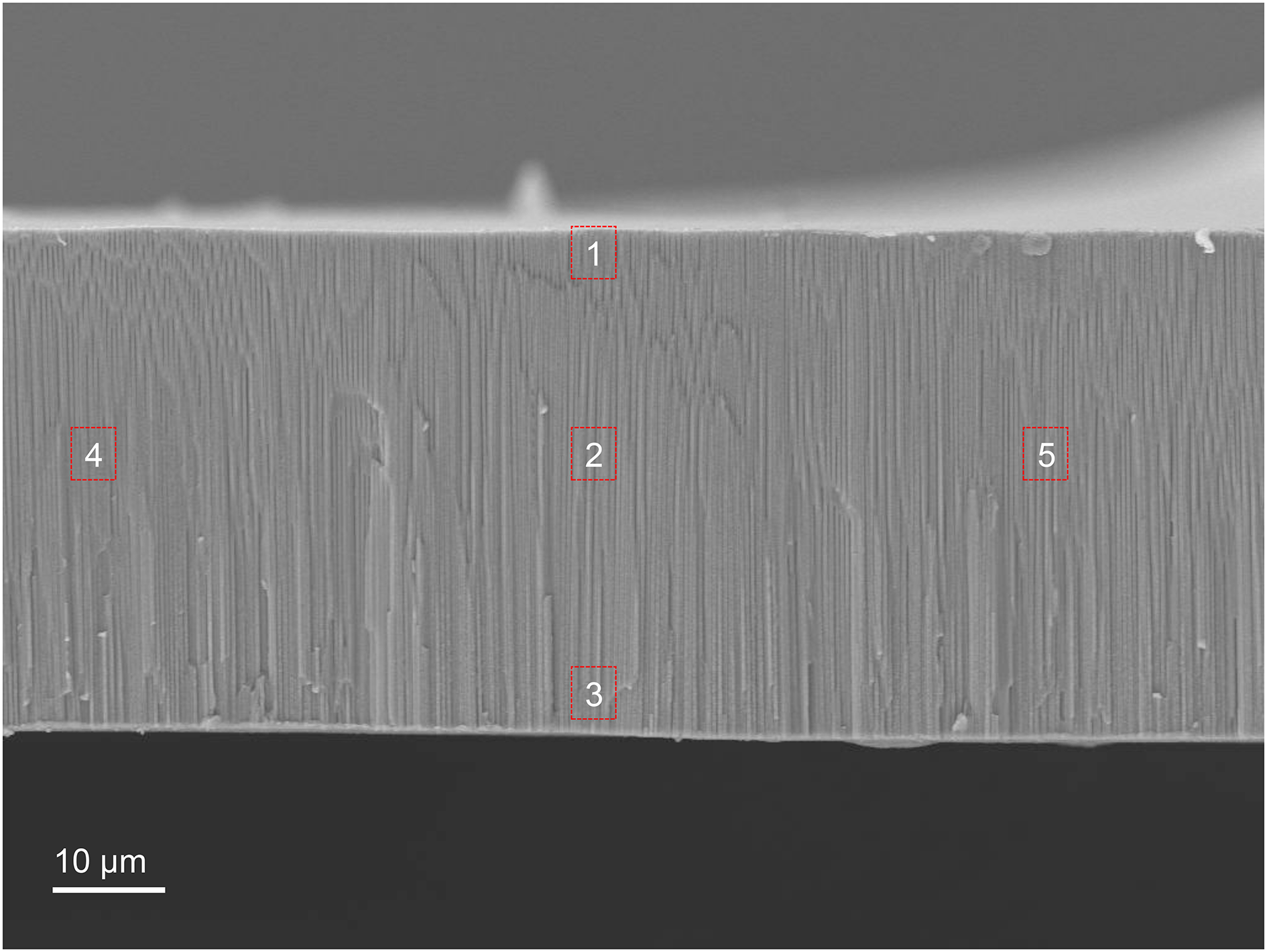


**Figure S26.** Cross-sectional SEM image of Cd_0.27_Zn_0.73_S/7 ML-ZnS QDs @AAO arrays.


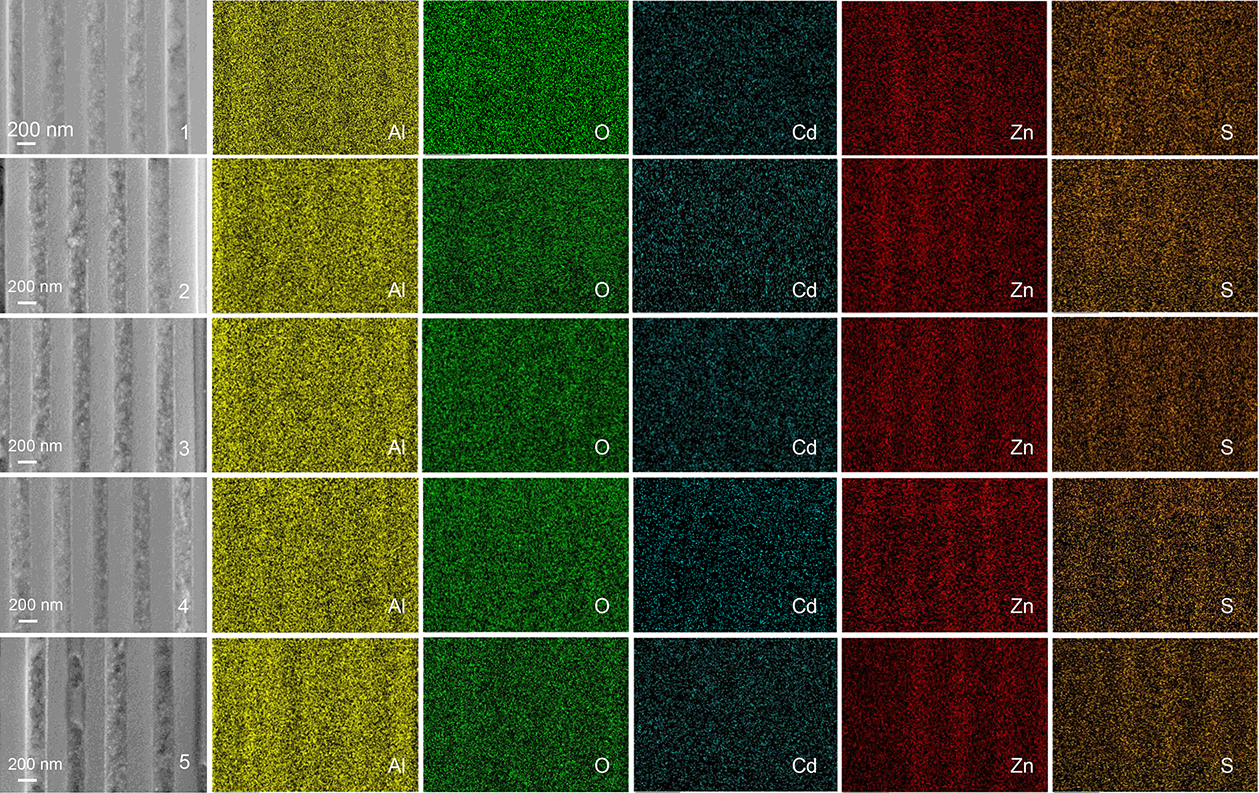


**Figure S27.** EDS elemental mapping images of five selected regions in the Cd_0.27_Zn_0.73_S/7 ML-ZnS QDs@AAO.


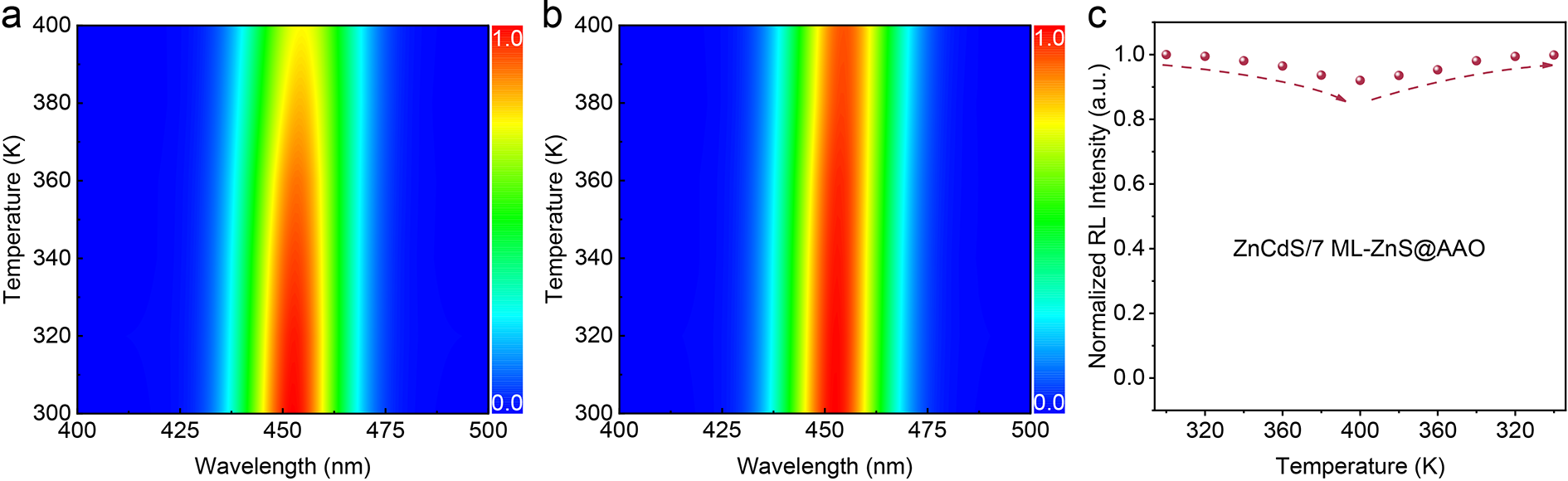


**Figure S28.** Temperature-dependent PL spectra of a) Cd_0.27_Zn_0.73_S/7 ML-ZnS core/shell QDs and b) Cd_0.27_Zn_0.73_S/7 ML-ZnS core/shell QDs@AAO measured from 300 to 400 K. c) Evolution of integral RL intensities of Cd_0.27_Zn_0.73_S/7 ML-ZnS core/shell QDs@AAO films at different temperatures.


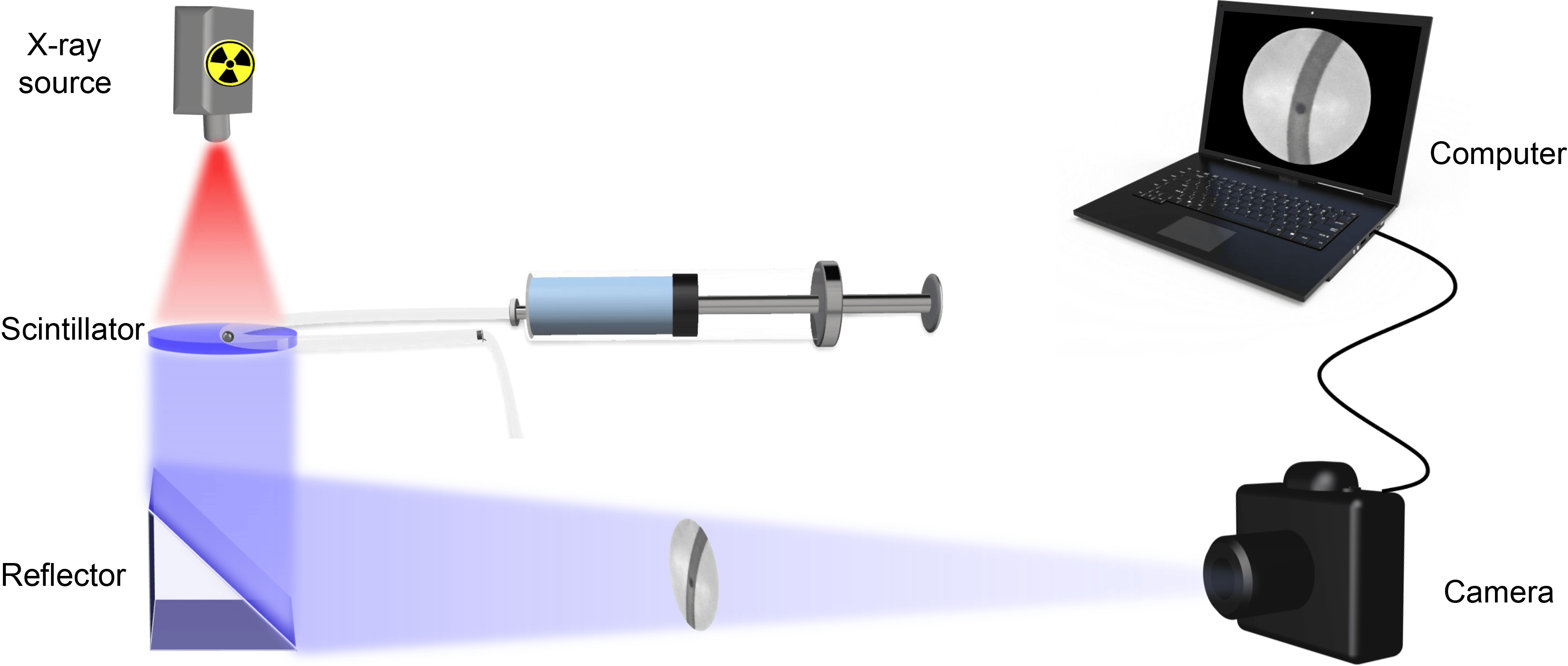


**Figure S29.** Self-built X-ray dynamic imaging system.


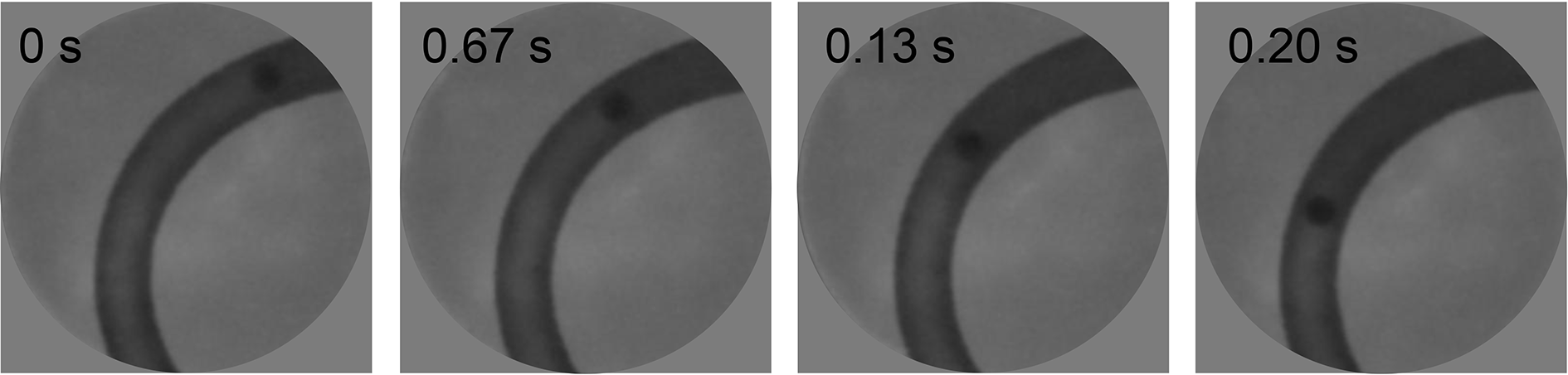


**Figure S30.** Dynamic X-ray imaging at 60 frames per second under continuous X-ray radiation (dose rate: 50.59 µGy s^‒1^; beam current: 19 µA).

**Table S1.** bioexponential fit parameters and average emission lifetimes for typical monitored wavelengths of Cd_0.27_Zn_0.73_S/y ML-ZnS core/shell QDs.

| Sample | τ_1_/ns | B_1_/% | τ_2_/ns | B_2_/% | τ_3_/ns | B_3_/% | τ_avg_/ns |
| --- | --- | --- | --- | --- | --- | --- | --- |
| Core | 8.96 | 11.67 | 56.08 | 34.30 | 336.94 | 54.03 | 202.31 |
| 2 ML | 15.08 | 44.37 | 119.05 | 55.63 | 0 | 0 | 72.92 |
| 3 ML | 13.46 | 46.95 | 91.95 | 53.05 | 0 | 0 | 55.10 |
| 4 ML | 12.34 | 44.85 | 86.82 | 55.15 | 0 | 0 | 53.42 |
| 5 ML | 8.48 | 55.54 | 33.30 | 44.16 | 0 | 0 | 19.52 |
| 7 ML | 8.07 | 80.05 | 41.88 | 19.95 | 0 | 0 | 14.82 |
| 8 ML | 10.89 | 73.47 | 48.31 | 26.53 | 0 | 0 | 20.81 |

**Table S2.** Fitting parameters of E_0_, α and β.

| sample | E_0_ (eV) | α (10^-4^ eV/K) | β (K) |
| --- | --- | --- | --- |
| CdZnS | 2.81 | 4.81 ± 0.63 | 261.69 ± 80.82 |
| CdZnS/ZnS | 2.88 | 4.16 ± 0.59 | 195.30 ± 79.89 |
| CdS bulk |  | 3.96 | 222 |
| ZnS bulk |  | 4.8 | 310 |
